# Supplementary material for: ‘God protects us from death through faith and science’: a qualitative study on the role of faith leaders in combating the COVID-19 pandemic and in building COVID-19 vaccine trust in Addis Ababa, Ethiopia
Source: BMJ Open. 2024 Apr 22;14(4):e071566. doi: 10.1136/bmjopen-2023-071566 (PMC11043698; doi:10.1136/bmjopen-2023-071566)
Supplement: Author's manuscript [file bmjopen-2023-071566.draft_revisions.pdf]

BMJ Open is committed to open peer review. As part of this commitment we make the peer review history of every article we publish publicly available.

When an article is published we post the peer reviewers' comments and the authors' responses online. We also post the versions of the paper that were used during peer review. These are the versions that the peer review comments apply to.

The versions of the paper that follow are the versions that were submitted during the peer review process. They are not the versions of record or the final published versions. They should not be cited or distributed as the published version of this manuscript.

BMJ Open is an open access journal and the full, final, typeset and author-corrected version of record of the manuscript is available on our site with no access controls, subscription charges or pay-per-view fees (http://bmjopen.bmj.com).

If you have any questions on BMJ Open's open peer review process please email info.bmjopen@bmj.com

BMJ Open

"God protects us from death through faith and science": A qualitative study on the role of the faith-leaders in building COVID-19 vaccine trust in Addis Ababa, Ethiopia

Journal:	*BMJ Open*
Manuscript ID	bmjopen-2023-071566
Article Type:	Original research
Date Submitted by the Author:	21-Jan-2023
Complete List of Authors:	Yibeltal, Kalkidan; Addis Continental Institute of Public Health, Department of Reproductive health and Population<br>Workneh, Firehiwot ; Addis Continental Institute of Public Health, Epidemiology and Biostatistics<br>Melesse, Hanna; Addis Continental Institute of Public Health, Department of Epidemiology and Biostatistics<br>Wolde, Habtamu; Independent Consultant<br>Kidane, Workagegnehu Tarekegn; Addis Continental Institute of Public Health, Department of Nutrition and Behavioral Sciences<br>Berhane, Yemane ; Addis Continental Institute of Public Health, Department of Epidemiology and Biostatistics<br>Herzig van Wees, Sibylle; Karolinska Institute, Department of Global Public Health
Keywords:	COVID-19, Public health < INFECTIOUS DISEASES, QUALITATIVE RESEARCH

SCHOLARONE™
Manuscripts

I, the Submitting Author has the right to grant and does grant on behalf of all authors of the Work (as defined in the below author licence), an exclusive licence and/or a non-exclusive licence for contributions from authors who are: i) UK Crown employees; ii) where BMJ has agreed a CC-BY licence shall apply, and/or iii) in accordance with the terms applicable for US Federal Government officers or employees acting as part of their official duties; on a worldwide, perpetual, irrevocable, royalty-free basis to BMJ Publishing Group Ltd ("BMJ") its licensees and where the relevant Journal is co-owned by BMJ to the co-owners of the Journal, to publish the Work in this journal and any other BMJ products and to exploit all rights, as set out in our licence.

The Submitting Author accepts and understands that any supply made under these terms is made by BMJ to the Submitting Author unless you are acting as an employee on behalf of your employer or a postgraduate student of an affiliated institution which is paying any applicable article publishing charge ("APC") for Open Access articles. Where the Submitting Author wishes to make the Work available on an Open Access basis (and intends to pay the relevant APC), the terms of reuse of such Open Access shall be governed by a Creative Commons licence – details of these licences and which Creative Commons licence will apply to this Work are set out in our licence referred to above.

Other than as permitted in any relevant BMJ Author's Self Archiving Policies, I confirm this Work has not been accepted for publication elsewhere, is not being considered for publication elsewhere and does not duplicate material already published. I confirm all authors consent to publication of this Work and authorise the granting of this licence.

For peer review only

"God protects us from death through faith and science": A qualitative study on the role of the faith-leaders in building COVID-19 vaccine trust in Addis Ababa, Ethiopia

Co-authors

Kalkidan Yibeltal - Department Reproductive Health and Population. Addis Continental Institute of Public Health

Firehiwot Workneh - Department of Epidemiology and Biostatistics. Addis Continental Institute of Public Health, Addis Ababa, Ethiopia

Hanna Melesse - Department of Epidemiology and Biostatistics. Addis Continental Institute of Public Health, Addis Ababa, Ethiopia

Habtamu Wolde – Independent consultant. Addis Ababa, Ethiopia

Workagegnehu Tarekegn Kidane - Department of Nutrition and Behavioral Sciences. Addis Continental Institute of Public Health, Addis Ababa, Ethiopia

Yemane Berhane - Department of Epidemiology and Biostatistics. Addis Continental Institute of Public Health, Addis Ababa, Ethiopia

Sibylle Herzig Van Wees - Department of Global Public Health, Karolinska Institutet, Stockholm, Sweden

Corresponding author: Kalkidan Yibeltal

Postal address- 26751/1000, Addis Ababa, Ethiopia

Email- kalkidanyibeltal@addiscontinental.edu.et

Word count - 3996

Abstract

Objective- This study aimed to explore faith leaders' perspectives on the COVID-19 vaccine and their role in building COVID-19 vaccine trust in Addis Ababa, Ethiopia.

Design- Qualitative study design with in-depth interview and thematic analysis was conducted.

Participants- Twenty-one faith leaders from the seven religious groups represented in the Inter-Religious Council of Ethiopia participated in the study.

Setting- The study was conducted in Addis Ababa, Ethiopia.

Results- The thematic analysis revealed three themes. Firstly, faith-leaders clearly understand the risks of COVID-19 pandemic, although most ascribed a spiritual meaning to the advent of the pandemic. The pandemic seriously affected the faith communities inflicting financial losses. Secondly, faith-leaders were important allies during the pandemic by effectively collaborating with government and health professionals in COVID-19 prevention activities and public health interventions using spiritual reasoning. They were actively informing the community about the importance of the COVID-19 vaccine where by many faith-leaders were publicly vaccinated to build trust in the vaccine and be role models. Thirdly, despite this they faced multiple questions from the congregation about the vaccine, including rumors.

Conclusions- This research showed that faith-leaders played crucial roles in encouraging vaccines use but limited in their persuasion power because of intense rumors and misinformation. Empowering faith-leaders with latest evidence on vaccines need to be given priority in the future.

Keywords: COVID-19, faith-leaders, vaccine, hesitancy, Ethiopia, religion

Strengths and limitations of this study

- Prolonged engagement with data was ensured as the co-authors conducted the interviews.
- Working with multiple coders aided in minimizing the subjective bias of any one researcher.
- Interviews were conducted among faith-leaders included in the structured inter-religious council, it did not directly interview those outside of this structure to capture their perception and role

For peer review only - http://bmjopen.bmj.com/site/about/guidelines.xhtml

Background

Equitable access to safe and effective vaccines is critical to control the spread of infectious pandemics such as COVID-19 and minimize the devastating adverse health outcomes (1). Achieving herd immunity using effective vaccines is critical to avert pandemic-related morbidity and mortality (2,3). Promoting the uptake of COVID-19 vaccines requires people's willingness to get vaccinated and ensuring proper communication of information through trusted sources to aid vaccine acceptance (4,5).

Ethiopia started the COVID-19 vaccination campaign in March 2021 with the Astra Zeneca vaccine, manufactured by the Serum Institute of India and supplied by the COVID-19 Vaccines Global Access (COVAX) initiative (6). The Ministry of Health launched a national ceremony to initiate the COVID-19 vaccine rollout in the presence of different stakeholders, including religious representatives. Initially, healthcare providers and older people with comorbidities were prioritized and provided with two doses of AstraZeneca vaccines (6,7). In the later stage, Johnson & Johnson vaccine, Sinopharm, and Pfizer vaccines were included in the campaign as they became available (7,8).

To bring about the intended effect of controlling the pandemic, vaccines need to be effectively utilized by the target population. Vaccine hesitancy, refers to refusal or delay to accept a vaccine despite the availability of the vaccination services is a major concern (9,10). WHO considers vaccine hesitancy one of the top 10 Global Health threats. Studies have shown high COVID-19 vaccine hesitancy among the Ethiopian population (11–13). Vaccine hesitancy related to COVID-19 has been driven by concerns about vaccine safety, efficacy, and side effects (14,15). Moreover, the slow rollout and politicization of vaccines in Africa has fueled rumors, mistrust, and conspiracy theories around these vaccines (16,17). Issues of mistrust towards the government and the private sector, reading misinformation on social media and the history of vaccination programs in a country have been further reasons for vaccine hesitancy among health workers and the population (15).

Global surveys show that religious factors are the third most commonly reported reason for vaccine hesitancy globally (18). Religious views can affect how people react to vaccinations, causing reactions like vaccine hesitancy, despite conveying medically sound and scientifically proven information/evidence (19). In the context of the COVID-19 pandemic, concerns have been raised

For peer review only - http://bmjopen.bmj.com/site/about/guidelines.xhtml

that religious communities may serve as basis for misinformation and unfounded theories that undermine the use of COVID-19 vaccines (20). Thus, faith-leaders' importance in addressing vaccine hesitancy has received increasing attention in global health research (21).

Ethiopia is a religious country where more than 97% of the people are religious and faith-leaders hold significant societal positions (22). It has been well documented that faith-leaders are embedded in society with high influencing positions, and often have considerable leverage with state and non-state powers due to the size of their constituencies. Therefore, engagement with faith-leaders is of critical importance as they are understood to be gatekeepers to local communities, with considerable influence on their communities' beliefs and behavior. Thus, this study aimed to explore faith-leaders' perspectives on the COVID-19 vaccine, and the role faith-leaders can play in building COVID-19 vaccine trust.

Methods

Study setting

This study was conducted in Addis Ababa, the capital city of Ethiopia. The main religions in Ethiopia are Orthodox Christianity (43% of women and 45% of men) and Islam (31% each of women and men). Twenty-three percent of women and 22% of men are Protestants followed by <1% Catholic and remaining small percentage of people following other faiths, including traditional faiths (23). The Inter-Religious Council of Ethiopian (IRCE) was established in 2010 to promote inter-religious harmony in Ethiopia (24). IRCE is represented at federal, regional, and district levels. The IRCE head office is located in Addis Ababa. This council is made of seven religious groups, and each has a representative in the head office.

Study design and aim

A qualitative study design using in-depth interviews was used to explore faith-leaders' perceptions of the COVID-19 vaccine and the role faith-leaders can play in building COVID-19 vaccine trust.

Study population

The participants of this study are faith-leaders selected from the seven religious groups represented in the IRCE. These are Ethiopian Orthodox Tewahido Church, Islam, 7th Day Adventist, Evangelical churches fellowship of Ethiopia, Ethiopian Kale Heywet church, Ethiopian Evangelical Church Mekane Eyesus and Ethiopian Catholic church.

For peer review only - http://bmjopen.bmj.com/site/about/guidelines.xhtml

Study sample and sampling procedure

Criteria-based purposive sampling was used to identify appropriate study participants. Through the inter-religious council, we identified the seven religious entities which constituted the IRCE. From these seven religions, informative individuals; one person per hierarchy, at three different positions of each religious entity; two faith-leaders at the head office with different positions and one from the religious institutions (Churches and Mosque) who have much closer contact with their congregation, were selected in consultation with each religion's head office highest representative body and were interviewed, which culminates to 21 participants.

Data collection procedures

In-depth interviews (IDI) were used for data collection. The themes of the interview guide were developed based on our background research. Themes included: faith-leaders understanding of COVID-19, rumors, and uncertainty around the COVID-19 vaccine, and role of faith-leaders related to the control of the pandemic. The research team developed the interview guide in English and then translated it into Amharic (the official language). Two research assistants with extensive experience in qualitative data collection were recruited to collect the data. The research assistants were trained for two days on the interview guides, the purpose of the study, and research ethics. Verbal consent was obtained prior to the interview. All interviews were audio recorded, and additional notes were taken during the in-depth interview. The IDIs were conducted in a private and quiet place, mainly in a place preferred by the participants, their offices most of the time. The interviews took 40 - 90 minutes and were conducted on dates and times convenient to the participants. COVID-19 prevention methods such as keeping physical distance, using hand sanitizers, and wearing masks were strictly implemented during the data gathering.

Data analysis

The research assistants transcribed all interviews verbatim and then translated from Amharic to English. Transcripts and translations were checked for accuracy and consistency by the two independent persons. Field notes taken during the IDIs were integrated into the transcription. KY, FW and SHvW conducted thematic qualitative analysis following Braun and Clarke's method (25). The researchers developed an intensive codebook and kept all audit trail records during the analysis, such as data reduction notes. The codebooks included definitions and example responses. Regular meetings, both in person and virtually, were made among the researchers to ensure consistency and validity. The Thematic analysis was done using Atlas.ti software version 7.5.16.

For peer review only - http://bmjopen.bmj.com/site/about/guidelines.xhtml

Working with multiple coders aids in minimizing the subjective bias of any one researcher. The coding process was continued until the data was exhausted. Then, the codes were categorized, and themes were created. The themes were developed and refined following several team meetings.

Patient and public involvement

No patient involved.

Ethical considerations

Participation in this study was entirely voluntary, and informed consent was obtained from all participants. The study procedures, benefits, and anticipated risks or discomforts of participating in the study and right to withdraw were explained. Principles of anonymity and confidentiality were applied to protect the participant's identity by assigning codes to participants. The audio recordings were destroyed after the completion and verification of the transcription and translations. The study was conducted in accordance with national ethical standards and the declaration of Helsinki (26).

For peer review only

For peer review only - http://bmjopen.bmj.com/site/about/guidelines.xhtml

Results

The thematic analysis revealed three themes and 15 categories, presented in table 1 and presented in this section. A category was created when a substantial number of codes or data extracts described that specific finding. When presenting the data in this section, we only use one data extract to describe the category.

Table 1 Themes and defined categories based on thematic analysis.

Theme	Category
Faith-leaders have a clear understanding of COVID-19, and they and their congregations have been seriously affected by the pandemic	Faith-leaders have a clear understanding of risks of COVID-19 pandemic
	Solitude in difficult times
	Financial loss for faith institutions
	Vulnerable groups are left behind
	Spiritual crisis
Faith leaders are important allies during a pandemic	Faith-leaders are trusted
	Faith-leaders collaborate with government and health professionals
	Faith-leaders engage in public health prevention using spiritual reasoning
	Faith-leaders trust medical science
	Faith-leaders are actively engaged in COVID-19 prevention methods
Faith-leaders intrigued by rumors and uncertainty around COVID-19 vaccine	Faith-leaders trust vaccines
	Rumors about vaccines are rampant in the community
	Spiritual rumors: 666
	Faith-leaders try to address concerns
	Faith-leaders are unsure how to respond to Rumors

Faith-leaders have a clear understanding of COVID-19, and they and their congregations have been seriously affected by the pandemic

Although most faith-leaders in this study ascribe a spiritual meaning to the advent of the COVID-19 pandemic, all faith-leaders interviewed in this study clearly understand the risk and severity of the COVID-19 pandemic. They are well informed and described in great detail the serious effects it has had on their religious practice and the members of their spiritual community. In the words

For peer review only - http://bmjopen.bmj.com/site/about/guidelines.xhtml

of a faith-leader: *"There is nothing it did not touch; it has affected our social life, economy, religious relations, our office, our work... We have lost so many people."* (IDI-12)

Faith-leaders describe the implications of solitude in difficult times, particularly during times of mourning and grief. Participants express profound sadness that they were unable to console individuals and support them during times of grief: *"We could not even comfort the families of the deceased after the funeral. We consider spiritual support as a psychosocial treatment, but we failed to offer that too."* (IDI-4)

Moreover, faith-leaders describe a spiritual crisis in their institutions whereby relationships between faith-leaders and those attending religious institutions have been harmed. This is particularly due to not allowing people participate in religious services, which led to conflicts in several religious institutions. Faith-leaders further describe a change in religious practice and organization, which has remained in practice till now:

> *It* [COVID-19] *even created another culture; older people and people with some medical conditions became too scared to come to our congregation even after we were allowed to gather again. In our religion/in, Christianity, the New Testament encourages and teaches togetherness to grow in holiness. So, it affected that core value.* (IDI-6)

Faith-leaders further describe the financial implications for their institutions. Due to the loss of participants in religious services, income diminished substantially. Businesses surrounding religious institutions were further negatively affected. This led to significant staff cuts at religious institutions. Moreover, it led to the closure of social activities and programs used to serve the needs of the poorest: *"With the limited money we got, we were only able to pay salaries church ministers; there was nothing left to give to those who are in need."* (IDI-4)

Despite these challenges, faith-leaders describe innovative approaches to countering challenges. This includes the use of social media to reach the community:

> *We tried to solve this and reach our members through technology by calling them over the phone and using the zoom application. Fortunately, we launched a Television program a month or two before the pandemic called Hope channel, thus we used that to preach and praise the Lord...* (IDI-14)

Active fundraising in the community was further organized to make up for the financial loss:

For peer review only - http://bmjopen.bmj.com/site/about/guidelines.xhtml

> *When everyone was told to stay at home, it was hard for our community because they have no money or food to eat especially during the fasting period. So, we have tried our best to provide them with food for the fasting period. We did that here in Addis Ababa and in some other areas to. For that, we have collected over 30 million birr from investors and distributed it to others.* (IDI-16)

Faith leaders are important allies during a pandemic

The second theme summarizes the work faith-leaders and their institutions did during the pandemic. To begin with, most faith-leaders collaborated closely with the Ministry of Health and health professionals to inform their congregations about the disease and preventive measures. The collaboration between the government and religious institutions appears to have been effective, and faith-leaders trusted the information and directives of the Ministry of Health: *"obeying the government is part of our faith"* (IDI-6).

In turn, faith-leaders consider themselves highly trusted within their community and congregations. They are consulted in times of hardship and on important family matters. They were also consulted about questions regarding the COVID-19 pandemic, particularly rumors about the origin of the pandemic and vaccines. There are numerous examples of how faith-leaders describe following directives, implementing recommended interventions (hand washing, mask use, social distancing), and actively informing the community about the importance of the COVID-19 vaccine.

> *There are questions forwarded to me; we often convey a message when we finish teaching in the church. That people should follow the instructions. In general, we tell them God loves us as we obey, wash your hands, we should respect each other, and the vaccine is also important you should take it. What we do here is critical; we are spiritual doctors.* (IDI-3)

Faith-leaders saw themselves as key in implementing measures to addressing the pandemic and proactively raising awareness to help implement directives.

> *We intensively created awareness, stressing that the vaccine is one of the major remedies for this disease. We assigned responsible persons and Imams who can transmit these messages during the five prayer times in every Mosque. Regarding*

For peer review only - http://bmjopen.bmj.com/site/about/guidelines.xhtml

> *awareness creation, we successfully changed people's understanding from wrong to right direction.* (IDI-16)

Faith-leaders describe how they use religious scriptures to promote guidelines to reduce transmission. This was particularly important when the community challenged them regarding these measures.

> *The Bible supports all the recommended preventive safety measures for COVID-19. So if parishioners come with such concerns, I will teach them this according to the Bible; and guide them in the right direction.* (IDI-2)

Several faith-leaders, regardless of the denomination, describe examples of how they use spiritual reasoning to address concerns: *…the Bible says that Luke, the Evangelist, was one of the writers of the Bible who had medical knowledge. Therefore, the doctrine of the Church does not contradict such wisdom.* (IDI-11) Almost all faith-leaders in this study describe a strong trust in science and use religious reasoning to support this. In the words of a faith-leader*: "God protects us from death through faith and science"* (IDI-21)

Faith-leaders are faced with rumors and uncertainty around COVID-19 vaccine

A major challenge described by faith-leaders was the rumors about the COVID-19 vaccine they encountered. This section summarizes faith-leaders' different engagement with these rumors. Despite the strong commitment of faith-leaders to implement preventative measures, they faced challenges in addressing rumors about vaccines. Most, but not all, faith-leaders in this study trust the COVID-19 vaccine. They describe their trust in science and the government and use religious reasoning to support their trust. Many faith-leaders were publicly vaccinated to build trust in the vaccine: *"I and other faith-leaders of different religions in Addis Ababa got vaccinated in public; we did that to be role models to our parishioners."* (IDI-13)

Participants describe numerous and consistent rumors encountered in the community. The COVID-19 vaccine was considered dangerous because it was sent from abroad and was therefore not trusted: *"Now Ethiopia is in disagreement with America as we all know, what if they send this vaccine to destroy us and attack us?"* (IDI-14). There were rumors that the COVID-19 vaccine represented 666 – the mark of the beast.

For peer review only - http://bmjopen.bmj.com/site/about/guidelines.xhtml

> *There is a religious teaching about 666*[1]*. I guess you have heard about that in every church. So, they say this vaccine is used to insert satanic 666 into our bodies. So, they say Satan makes the vaccine so this is satanic teaching. They say participating in this is like partnering with Satan.* (IDI-13)

Moreover, a repeated concern that faith-leaders faced, and some addressed themselves was the effect of the COVID-19 vaccine on fertility. Faith-leaders describe how rumors affected their own beliefs and they describe challenges in addressing these questions:

> *..it is rumored that getting vaccinated can cause infertility. They also say the developed countries fabricate this disease to minimize the population of highly populated countries like African countries. There were such kinds of many questions. It was tough to answer such questions.* (IDI-16)

Few faith-leaders actively counter the rumors but those who do use spiritual reasoning to do so. For example:

> *When they say it will make you infertile, I will say, are you a prophet? Have you been given the gift of prophesying? So this shows there is awareness problem, all this due to lack of awareness, that's how I see it.* (IDI-3)

However, several faith-leaders describe that they themselves got uncertain about the vaccine as a result of the rumors: "*It's best to try to understand what they want to say, I don't have evidence it might be what they said or it may not. So the main thing is believing.*" (IDI-21)

Moreover, some described that they did not feel in a position to address those concerns. They were more comfortable preaching about all other public health prevention measures than the vaccine.

> *One of our [faith-leaders'] tasks is to educate the community on preventing these outbreaks as health professionals recommended. Teaching about the disease/ pandemic is comfortable, but I cannot say anything about specific vaccines.* (IDI-11)

---

[1] *According to most manuscripts of the New Testament and in English translations of the Bible, the number of the beast is six hundred sixty-six. (Book of Revelation 13:15-18, Wikipedia)*

For peer review only - http://bmjopen.bmj.com/site/about/guidelines.xhtml

Discussion

This qualitative study explored faith-leaders' perception of the COVID-19 vaccine and their role in building COVID-19 vaccine trust in Addis Ababa. The findings of our study showed that most faith-leaders included in this study clearly understand the risks associated with COVID-19. Due to the advent of the COVID-19 pandemic, faith-leaders and their congregations have faced solitude in difficult times. Additionally, religious institutions have encountered significant financial and spiritual crises. Our study also revealed that faith-leaders were actively engaged in pandemic measures and held strong beliefs in the medical sciences, including vaccines. However, despite the high trust in vaccines and medical science, faith-leaders struggled to address COVID-19 vaccine rumors in their communities.

This study indicated that faith-leaders were well-informed about the risks of COVID-19. This could be due to good access to sources regarding COVID-19 pandemic due to their networks, mass media, and social media exposure. Our findings disclosed that due to the COVID-19 pandemic, vulnerable groups, faith-leaders, and their congregations have faced solitude under challenging times and encountered significant financial and spiritual crises. This finding is in line with the findings of a qualitative study conducted in Ghana, which showed that the COVID-19 pandemic caused economic challenges to the congregants, a decline in spiritual life, loss of fellowship and community(27). Another qualitative study conducted on United Methodist pastors in one of the two Annual Conferences in North Carolina Pastors reported that COVID-19 fundamentally unsettled routine works of the ministry(28).

This study also identified innovative approaches used by faith-leaders to tackle the challenges. This includes the use of social media to reach the community and active fundraising in the community to make up for the financial loss. A qualitative study on Ghanaian Christian Leaders also showed that as a response to the ban, most participants in their study explicitly reported that they moved at least some activities online, which included conventional activities, such as posting prayer topics and live streaming of services for audiences who wished to view them synchronously (29). Similarly, religious authorities in Uganda played a critical role in delivering public health messages on COVID-19 risk communication using the web (16).

According to a recent systematic review that assessed religious communities' role, religion has acted as an important platform for inter-sectoral collaboration with science and government to

For peer review only - http://bmjopen.bmj.com/site/about/guidelines.xhtml

combat COVID-19 (30). National, regional and local faith-leaders have high levels of influence and community-organizing capabilities. Faith-leaders are effective messengers endorsing vaccination compared to other potential messengers (31). They can help frame approaches that will make them more likely to be accepted in their communities. Governments should build trust with faith-leaders and integrate them into planning, decision making and implementation at every level of their COVID-19 response (32). The findings in this study also showed that there was a strong collaboration between the government and religious institutions which appeared to have been effective, and faith-leaders trusted the information and directives of the Ministry of Health. This might be due to closeness of the religious institutions to each other and the government through a well-founded and organized inter-religious council that collaborates with the government on different national agendas (24). Besides, all faith-leaders interviewed for this study consider themselves highly trusted within their community and congregations. They were consulted about questions regarding the COVID-19 pandemic, particularly rumors about the origin of the pandemic and vaccines. Due to that, most of the study participants saw themselves as key actors in implementing the safety measures and in proactively raising awareness to help implement directives to address the pandemic. Since faith-leaders are often highly influential community leaders who are 'listened to by the community members, they often underutilize potential for catalyzing change(21). In line with this finding, a qualitative case study conducted in Indonesia showed that the faith-leaders supported the health directives designed to reduce high transmission risk (33). All the scholars and faith-leaders stated that funerals, according to the health protocols issued by the authorities, should be conducted considering potential disease transmission. Some Islamic organizations have developed their guidelines for COVID-19 by modifying the religious values of certain institutions to prevent the spread of infectious disease(34).

This study revealed that regardless of the denomination, almost all faith-leaders who participated in this study said they have trust in science and used religious scriptures and spiritual reasoning to promote guidelines to reduce the transmission of COVID-19. A study has also shown that many faith-leaders see the vaccine as a message of hope, and Pope Frances also indicated that it is a moral obligation of everyone to be vaccinated (35). Our study participants also added that many faith-leaders were publicly vaccinated to build trust in the vaccine. This was particularly important when the community challenged them regarding these measures. In agreement with that, findings of qualitative study conducted in Leeds, United Kingdom, also revealed that faith leaders could

For peer review only - http://bmjopen.bmj.com/site/about/guidelines.xhtml

play a very important role in the health behavior of their congregants (36). Faith-leaders can influence health behavior not only on the individual level but also on a socio-cultural and environmental level. They exert such influence through several mediators, including scriptural power, social influence and by serving as a role models (37). Our results showed that most of the interviewed faith-leaders faced numerous and consistent rumors encountered in the community and faith-leaders themselves were affected by the rumors they heard about COVID-19 vaccine and faced challenges in addressing such questions.

The African center for strategic studies reported preponderance of COVID-19 vaccine myths is causing many Africans to forego vaccinations at a time when new, more transmissible coronavirus variants are spreading across the continent (38). The report indicated several myths including live viruses are injected to cause death after vaccination, the vaccine causes infertility by altering the DNA to reduce Africa's population and other serious side effects that are worse than contracting the virus, and some claim that vaccines are a cover to implant traceable microchips implant traceable microchips (38). Such myths and similar rumors may lead to COVID-19 vaccine hesitancy in the general community. In line with these findings, a population based online survey conducted in Ethiopia showed that only 31.4% of the total participants were willing to receive the COVID-19 vaccine (39). One of the strategies to counter vaccine hesitancy is to follow a multisectoral approach that involves the collaboration between various stakeholders, such as government, private companies, religious groups, and other agencies, to leverage the knowledge, expertise, and resources, thereby enabling the creation of longstanding public trust of vaccines (35,36). Given the importance of faith-leaders and their trust in science, it appears indispensable to equip faith-leaders with the knowledge and skills to address rumors.

Limitations of the study can lay in the fact that we only conducted the interview among faith-leaders included in the structured inter-religious council and did not directly interview that outside of this structure to capture their perception and role. Although the findings cannot be generalized, inferential generalizations can be drawn, and findings may be relevant for other similar settings.

Conclusion

Faith-leaders have played an important role in mitigating the COVID-19 pandemic in Ethiopia. Faith-leaders have followed and implemented government directives and also promoted the vaccine, further amplifying their important alliance in public health. However, they have been

For peer review only - http://bmjopen.bmj.com/site/about/guidelines.xhtml

challenged with different rumors about the vaccine, which they struggled to address as they were not equipped to do so. Thus, empowering faith-leaders to address rumors about new vaccines could have significant public health implications for ongoing and future pandemics.

Contributorship statement – YB, SHvW, FW and KY designed the study. HM & HW conducted the interviews, transcriptions and translations. KY, FW, HM, WT and SHvW involved in the coding and conducted the analysis. All authors collaborated on the writing of the manuscript and final draft was written by KY & FW. All authors have read and approved the final manuscript.

Competing Interests - No conflicts of interests to declare.

Funding – The study is funded by the Addis Continental Institute of Public Health, Ethiopia. SHvW is the recipient of a Postdoc grant awardee by FORTE (STY396 2021/0001) in Sweden.

Data sharing statement – No additional data available

Ethics approval- Ethical clearance was obtained from the Institutional Review Board (IRB) of Addis Continental Institute of Public Health (ACIPH/IRB/010/2021).

Acknowledgments – We would like to acknowledge the inter-religious council of Ethiopia for their collaboration for the study.

For peer review only - http://bmjopen.bmj.com/site/about/guidelines.xhtml

Reference

1. Legge DG, Kim S. Equitable Access to COVID-19 Vaccines: Cooperation around Research and Production Capacity Is Critical. J Peace Nucl Disarm. 2021 Mar 5;4(sup1):73–134.

2. Bardosh K, Figueiredo A de, Gur-Arie R, Jamrozik E, Doidge J, Lemmens T, et al. The unintended consequences of COVID-19 vaccine policy: why mandates, passports and restrictions may cause more harm than good. BMJ Glob Health. 2022 May 1;7(5):e008684.

3. Pilkington V, Keestra SM, Hill A. Global COVID-19 Vaccine Inequity: Failures in the First Year of Distribution and Potential Solutions for the Future. Front Public Health [Internet]. 2022 [cited 2022 Nov 5];10. Available from: https://www.frontiersin.org/articles/10.3389/fpubh.2022.821117

4. Accelerating COVID-19 Vaccine Deployment : Removing Obstacles to Boost Coverage Levels and Protect Those at High-risk [Internet]. [cited 2022 Nov 5]. Available from: https://documents.worldbank.org/en/publication/documents-reports/documentdetail/099352004212214271/idu020e3b11b090ce0494d0aaf50e9e074fc8

5. Collaboration in times of crisis: A study on COVID-19 vaccine R&D partnerships - PMC [Internet]. [cited 2022 Nov 5]. Available from: https://www.ncbi.nlm.nih.gov/pmc/articles/PMC8410639/

6. Ethiopia introduces COVID-19 vaccine in a national launching ceremony [Internet]. WHO | Regional Office for Africa. [cited 2022 Nov 9]. Available from: https://www.afro.who.int/news/ethiopia-introduces-covid-19-vaccine-national-launching-ceremony

7. COVAX | MINISTRY OF HEALTH - Ethiopia [Internet]. [cited 2022 Nov 9]. Available from: https://www.moh.gov.et/site/covax

8. The Ministry of Health has received 10 million doses of covid 19 vaccine from the Chinese government. | MINISTRY OF HEALTH - Ethiopia [Internet]. [cited 2022 Nov 9]. Available from: https://www.moh.gov.et/site/node/352

9. Mesch GS, Schwirian KP. Vaccination hesitancy: fear, trust, and exposure expectancy of an Ebola outbreak. Heliyon. 2019 Jul 18;5(7):e02016.

10. Sallam M. COVID-19 Vaccine Hesitancy Worldwide: A Concise Systematic Review of Vaccine Acceptance Rates. Vaccines. 2021 Feb 16;9(2):160.

11. Scheres J, Kuszewski K. The Ten Threats to Global Health in 2018 and 2019. A welcome and informative communication of WHO to everybody. Zdr Publiczne Zarządzanie. 2019;17(1):2–8.

12. COVID-19 vaccine hesitancy in Addis Ababa, Ethiopia: a mixed-method study | BMJ Open [Internet]. [cited 2022 Nov 5]. Available from: https://bmjopen.bmj.com/content/12/5/e052432.citation-tools

For peer review only - http://bmjopen.bmj.com/site/about/guidelines.xhtml

13. Rikitu Terefa D, Shama AT, Feyisa BR, Ewunetu Desisa A, Geta ET, Chego Cheme M, et al. COVID-19 Vaccine Uptake and Associated Factors Among Health Professionals in Ethiopia. Infect Drug Resist. 2021 Dec 19;14:5531–41.

14. Solís Arce JS, Warren SS, Meriggi NF, Scacco A, McMurry N, Voors M, et al. COVID-19 vaccine acceptance and hesitancy in low- and middle-income countries. Nat Med. 2021 Aug;27(8):1385–94.

15. Biswas MdR, Alzubaidi MS, Shah U, Abd-Alrazaq AA, Shah Z. A Scoping Review to Find Out Worldwide COVID-19 Vaccine Hesitancy and Its Underlying Determinants. Vaccines. 2021 Oct 25;9(11):1243.

16. Ekwebelem OC, Onyeaka H, Yunusa I, Miri T, Onwuneme YM, Eunice AT, et al. Do we trust the government? Attributes of COVID-19 vaccine hesitancy and acceptance in Nigeria. AIMS Med Sci. 2022;9(2):268–82.

17. Dereje N, Tesfaye A, Tamene B, Alemeshet D, Abe H, Tesfa N, et al. COVID-19 vaccine hesitancy in Addis Ababa, Ethiopia: a mixed-method study. BMJ Open. 2022 May;12(5):e052432.

18. Effects of Faith Actor Engagement on the Uptake and Coverage of Immunization in Low- and Middle-Income Countries [Internet]. USAID MOMENTUM. [cited 2021 Oct 29]. Available from: https://usaidmomentum.org/resource/vaccine-hesitancy-report/

19. The role of religiosity in COVID-19 vaccine hesitancy - PubMed [Internet]. [cited 2022 Oct 31]. Available from: https://pubmed.ncbi.nlm.nih.gov/34080617/

20. COVID-19 Information Sources and Misinformation by Faith Community - PubMed [Internet]. [cited 2022 Oct 31]. Available from: https://pubmed.ncbi.nlm.nih.gov/35634989/

21. Sleeping-Giants-mobilising-faith-leaders-as-agents-of-change-FINAL-DRAFT-2.pdf [Internet]. [cited 2021 Nov 2]. Available from: https://jliflc.com/wp-content/uploads/2017/11/Sleeping-Giants-mobilising-faith-leaders-as-agents-of-change-FINAL-DRAFT-2.pdf

22. Ethiopia Summary and Statistical Report of the 2007 Population and Housing Census - Ethiopia Summary and Statistical Report of the 2007 Population and Housing Census.pdf - openAFRICA [Internet]. [cited 2022 Nov 5]. Available from: https://africaopendata.org/dataset/ethiopia-summary-and-statistical-report-of-the-2007-population-and-housing-census/resource/1af0860e-2fa6-4711-b9a7-53ddb04e721f?view_id=08c5913c-0623-4358-bae8-cd64de358071

23. Central Statistical Agency. Ethiopia Demographic and Health Survey 2016 [Internet]. Addis Ababa, Ethiopia; [cited 2022 Dec 13]. Available from: https://dhsprogram.com/pubs/pdf/FR328/FR328.pdf

24. Inter-Religious Council of Ethiopia | URI [Internet]. [cited 2022 Nov 9]. Available from: https://www.uri.org/who-we-are/cooperation-circle/inter-religious-council-ethiopia

For peer review only - http://bmjopen.bmj.com/site/about/guidelines.xhtml

25. Braun V, Clarke V. Using thematic analysis in psychology. Qual Res Psychol. 2006 Jan 1;3(2):77–101.

26. World Medical Association Declaration of Helsinki: Ethical Principles for Medical Research Involving Human Subjects. JAMA. 2013 Nov 27;310(20):2191.

27. Osei-Kuffour F, Kwasi Peprah W, Marfo Sarfo D, Osei Yeboah B. COVID-19 Impact on Church Cash Inflows in Ghana as Moderated by Location Profile. Appl Finance Account. 2022 Oct 13;8(1):6.

28. Johnston EF, Eagle DE, Headley J, Holleman A. Pastoral Ministry in Unsettled Times: A Qualitative Study of the Experiences of Clergy During the COVID-19 Pandemic. Rev Relig Res. 2022 Jun 1;64(2):375–97.

29. Adams G, Osei-Tutu A, Affram AA, Phillips-Kumaga L, Dzokoto VAA. Implications of COVID-19 Innovations for Social Interaction: Provisional Insights From a Qualitative Study of Ghanaian Christian Leaders. Front Psychol [Internet]. 2022 [cited 2022 Oct 30];13. Available from: https://www.frontiersin.org/articles/10.3389/fpsyg.2022.647979

30. Lee M, Lim H, Xavier MS, Lee EY. "A Divine Infection": A Systematic Review on the Roles of Religious Communities During the Early Stage of COVID-19. J Relig Health. 2022 Feb 1;61(1):866–919.

31. Viskupic F, Wiltse D. The Messenger Matters: Religious Leaders and Overcoming COVID-19 Vaccine Hesitancy. :10.

32. Tony-Blair-Institute-Working-With-Religious-Leaders-to-Support-Public-Health-Measures.pdf [Internet]. [cited 2022 Oct 31]. Available from: https://docs.google.com/viewerng/viewer?url=https://jliflc.com/wp-content/uploads/2020/05/Tony-Blair-Institute-Working-With-Religious-Leaders-to-Support-Public-Health-Measures.pdf

33. Funeral Processes During the COVID-19 Pandemic: Perceptions Among Islamic Religious Leaders in Indonesia - PMC [Internet]. [cited 2022 Nov 5]. Available from: https://www.ncbi.nlm.nih.gov/pmc/articles/PMC8409696/

34. Nurhayati N, Purnama TB. Funeral Processes During the COVID-19 Pandemic: Perceptions Among Islamic Religious Leaders in Indonesia. J Relig Health. 2021 Oct;60(5):3418–33.

35. Galang JRF. Science and religion for COVID-19 vaccine promotion. J Public Health Oxf Engl. 2021 Apr 19;fdab128.

36. Heward-Mills NL, Atuhaire C, Spoors C, Pemunta NV, Priebe G, Cumber SN. The role of faith leaders in influencing health behaviour: a qualitative exploration on the views of Black African christians in Leeds, United Kingdom. Pan Afr Med J [Internet]. 2018 Jul 6 [cited 2022 Nov 5];30(1). Available from: https://www.ajol.info/index.php/pamj/article/view/209768

For peer review only

37. Heward-Mills NL, Atuhaire C, Spoors C, Pemunta NV, Priebe G, Cumber SN. The role of faith leaders in influencing health behaviour: a qualitative exploration on the views of Black African christians in Leeds, United Kingdom. Pan Afr Med J [Internet]. 2018 Jul 6 [cited 2022 Oct 30];30(1). Available from: https://www.ajol.info/index.php/pamj/article/view/209768

38. Dispelling COVID Vaccine Myths in Africa [Internet]. Africa Center for Strategic Studies. [cited 2022 Nov 1]. Available from: https://africacenter.org/spotlight/dispelling-covid-vaccine-myths-in-africa/

39. Belsti Y, Gela YY, Akalu Y, Dagnew B, Getnet M, Seid MA, et al. <p>Willingness of Ethiopian Population to Receive COVID-19 Vaccine</p>. J Multidiscip Healthc. 2021 May 28;14:1233–43.

40. Corpuz JCG. Multisectoral Approach on COVID-19 vaccination: a proposed solution on vaccine hesitancy. J Public Health Oxf Engl. 2021 Mar 18;fdab085.

41. Dhama K, Sharun K, Tiwari R, Dhawan M, Emran TB, Rabaan AA, et al. COVID-19 vaccine hesitancy - reasons and solutions to achieve a successful global vaccination campaign to tackle the ongoing pandemic. Hum Vaccines Immunother. 2021 Oct 3;17(10):3495–9.

For peer review only

For peer review only - http://bmjopen.bmj.com/site/about/guidelines.xhtml

Consolidated criteria for reporting qualitative studies (COREQ): 32-item checklist

Please indicate in which section each item has been reported in your manuscript. If you do not feel an item applies to your manuscript, please enter N/A.

For further information about the COREQ guidelines, please see Tong *et al.*, 2017: https://doi.org/10.1093/intqhc/mzm042

No.	Item	Description	Page #
**Domain 1: Research team and reflexivity**			
Personal characteristics			
1.	Interviewer/facilitator	Which author/s conducted the interview or focus group?	15
2.	Credentials	What were the researcher's credentials? *E.g. PhD, MD*	PhD, MPH, MD
3.	Occupation	What was their occupation at the time of the study?	Professor, Post doctoral researcher, Assistant Professors, Lecturer and research associate
4.	Gender	Was the researcher male or female?	Kalkidan, Firehiwot, Workagnehu, Hanna are females whereas Yemane and Habtamu are males
5.	Experience and training	What experience or training did the researcher have?	Minimum of 5 years and above experience on research and training
Relationship with participants			
6.	Relationship established	Was a relationship established prior to study commencement?	N/A
7.	Participant knowledgeof the interviewer	What did the participants know about the researcher? *E.g. Personal goals, reasons for doing the research*	N/A
8.	Interviewer characteristics	What characteristics were reported about the interviewer/facilitator? *E.g. Bias, assumptions, reasons and interests in the research topic*	N/A
**Domain 2: Study design**			
Theoretical framework			
9.	Methodological orientation and theory	What methodological orientation was stated to underpin the study? *E.g. grounded theory, discourse analysis, ethnography, phenomenology, content analysis*	5
Participant selection			
10.	Sampling	How were participants selected? *E.g. purposive, convenience, consecutive, snowball*	5
11.	Method of approach	How were participants approached? *E.g. face-to-face, telephone, mail, email*	5
12.	Sample size	How many participants were in the study?	5
13.	Non-participation	How many people refused to participate or dropped out? What were the reasons for this?	N/A

For peer review only

Setting			
14.	Setting of data collection	Where was the data collected? *E.g. home, clinic, workplace*	5
15.	Presence of non-participants	Was anyone else present besides the participants and researchers?	5

For peer review only

16.	Description of sample	What are the important characteristics of the sample? *E.g. demographic data, date*	5
Data collection			
17.	Interview guide	Were questions, prompts, guides provided by the authors? Was it pilot tested?	5
18.	Repeat interviews	Were repeat interviews carried out? If yes, how many?	N/A
19.	Audio/visual recording	Did the research use audio or visual recording to collect the data?	5
20.	Field notes	Were field notes made during and/or after the interview or focus group?	5
21.	Duration	What was the duration of the interviews or focus group?	5
22.	Data saturation	Was data saturation discussed?	No
23.	Transcripts returned	Were transcripts returned to participants for comment and/or correction?	N/A
**Domain 3: analysis and findings**			
Data analysis			
24.	Number of data coders	How many data coders coded the data?	15
25.	Description of the coding tree	Did authors provide a description of the coding tree?	5 & 7
26.	Derivation of themes	Were themes identified in advance or derived from the data?	5
27.	Software	What software, if applicable, was used to manage the data?	5
28.	Participant checking	Did participants provide feedback on the findings?	N/A
Reporting			
29.	Quotations presented	Were participant quotations presented toillustrate the themes / findings? Was each quotation identified? *E.g. Participant number*	8,9,10 & 11
30.	Data and findings consistent	Was there consistency between the data presented and the findings?	12, 13 & 14
31.	Clarity of major themes	Were major themes clearly presented in the findings?	7
32.	Clarity of minor themes	Is there a description of diverse cases or discussion of minor themes?	N/A

When submitting your manuscript via the online submission form, please upload the completed checklist as a Figure/supplementary file.

If you would like this checklist to be included alongside your article, we ask that you upload the completed checklist to an online repository and include the guideline type, name of the repository, DOI and license in the *Data availability* section of your manuscript.

Developed from: Allison Tong, Peter Sainsbury, Jonathan Craig, Consolidated criteria for reporting qualitative research (COREQ): a 32-item checklist for interviews and focus groups, International Journal for Quality in Health Care, Volume 19, Issue 6, December 2007, Pages 349–357, https://doi.org/10.1093/intqhc/mzm042

For peer review only

"God protects us from death through faith and science": A qualitative study on the role of the faith-leaders in in combating the COVID-19 pandemic and in building COVID-19 vaccine trust in Addis Ababa, Ethiopia.

Journal:	*BMJ Open*
Manuscript ID	bmjopen-2023-071566.R1
Article Type:	Original research
Date Submitted by the Author:	08-Dec-2023

Complete List of Authors:	Yibeltal, Kalkidan; Addis Continental Institute of Public Health, Department of Reproductive health and Population<br>Workneh, Firehiwot ; Addis Continental Institute of Public Health, Epidemiology and Biostatistics<br>Melesse, Hanna; Addis Continental Institute of Public Health, Department of Epidemiology and Biostatistics<br>Wolde, Habtamu; Independent Consultant<br>Kidane, Workagegnehu Tarekegn; Addis Continental Institute of Public Health, Department of Nutrition and Behavioral Sciences<br>Berhane, Yemane ; Addis Continental Institute of Public Health, Department of Epidemiology and Biostatistics<br>Herzig van Wees, Sibylle; Karolinska Institute, Department of Global Public Health

**Primary Subject Heading**:	Public health
Secondary Subject Heading:	Global health
Keywords:	COVID-19, Public health < INFECTIOUS DISEASES, QUALITATIVE RESEARCH

SCHOLARONE™
Manuscripts

I, the Submitting Author has the right to grant and does grant on behalf of all authors of the Work (as defined in the below author licence), an exclusive licence and/or a non-exclusive licence for contributions from authors who are: i) UK Crown employees; ii) where BMJ has agreed a CC-BY licence shall apply, and/or iii) in accordance with the terms applicable for US Federal Government officers or employees acting as part of their official duties; on a worldwide, perpetual, irrevocable, royalty-free basis to BMJ Publishing Group Ltd ("BMJ") its licensees and where the relevant Journal is co-owned by BMJ to the co-owners of the Journal, to publish the Work in this journal and any other BMJ products and to exploit all rights, as set out in our licence.

The Submitting Author accepts and understands that any supply made under these terms is made by BMJ to the Submitting Author unless you are acting as an employee on behalf of your employer or a postgraduate student of an affiliated institution which is paying any applicable article publishing charge ("APC") for Open Access articles. Where the Submitting Author wishes to make the Work available on an Open Access basis (and intends to pay the relevant APC), the terms of reuse of such Open Access shall be governed by a Creative Commons licence – details of these licences and which Creative Commons licence will apply to this Work are set out in our licence referred to above.

Other than as permitted in any relevant BMJ Author's Self Archiving Policies, I confirm this Work has not been accepted for publication elsewhere, is not being considered for publication elsewhere and does not duplicate material already published. I confirm all authors consent to publication of this Work and authorise the granting of this licence.

For peer review only

"God protects us from death through faith and science": A qualitative study on the role of the faith-leaders in in combating the COVID-19 pandemic and in building COVID-19 vaccine trust in Addis Ababa, Ethiopia.

Co-authors

Kalkidan Yibeltal - Department Reproductive Health and Population. Addis Continental Institute of Public Health

Firehiwot Workneh - Department of Epidemiology and Biostatistics. Addis Continental Institute of Public Health, Addis Ababa, Ethiopia

Hanna Melesse - Department of Epidemiology and Biostatistics. Addis Continental Institute of Public Health, Addis Ababa, Ethiopia

Habtamu Wolde – Independent consultant. Addis Ababa, Ethiopia

Workagegnehu Tarekegn Kidane - Department of Nutrition and Behavioral Sciences. Addis Continental Institute of Public Health, Addis Ababa, Ethiopia

Yemane Berhane - Department of Epidemiology and Biostatistics. Addis Continental Institute of Public Health, Addis Ababa, Ethiopia

Sibylle Herzig Van Wees - Department of Global Public Health, Karolinska Institutet, Stockholm, Sweden

Corresponding author: Kalkidan Yibeltal

Postal address- 26751/1000, Addis Ababa, Ethiopia

Email- kalkidanyibeltal@addiscontinental.edu.et

Word count - 4043

Abstract

Objective- This study explored faith leaders' perspectives on the COVID-19 vaccine and their role in building COVID-19 vaccine trust in Addis Ababa, Ethiopia.

Design- A qualitative study with in-depth interviews and thematic analysis was conducted.

Participants- Twenty-one faith-leaders from the seven religious groups represented in the Inter-Religious Council of Ethiopia participated in the study.

Setting- The study was conducted in Addis Ababa, Ethiopia.

Results- The thematic analysis revealed three themes. Firstly, faith-leaders were aware of the risks of the COVID-19 pandemic, although most ascribed a spiritual meaning to the advent of the pandemic. The pandemic seriously affected the faith communities, inflicting financial losses. Secondly, faith-leaders were essential allies during the pandemic by effectively collaborating with government and health professionals in COVID-19 prevention activities and public health interventions using spiritual reasoning. They were actively informing the community about the importance of the COVID-19 vaccine, where many faith-leaders were publicly vaccinated to build trust in the vaccine and be role models. Thirdly, despite this, they faced multiple questions from the congregation about the vaccine, including rumors.

Conclusions- This research showed that faith-leaders played crucial roles in encouraging vaccine use but were limited in their persuasion power because of intense rumors and misinformation. Empowering faith-leaders with the latest vaccine evidence needs to be prioritized in the future.

Keywords: COVID-19, faith-leaders, vaccine, hesitancy, Ethiopia, religion

Strengths and limitations of this study

- Prolonged engagement with data was ensured as the co-authors conducted the interviews.
- Working with multiple coders aided in minimizing the subjective bias of any researchers.
- Interviews were conducted among faith-leaders included in the structured inter-religious council; it did not directly interview those outside of this structure to capture their perception and role

Background

For peer review only - http://bmjopen.bmj.com/site/about/guidelines.xhtml

Equitable access to safe and effective vaccines is critical to control the spread of infectious pandemics such as COVID-19 and minimize the devastating adverse health outcomes (1). Achieving herd immunity using effective vaccines is essential to avert pandemic-related morbidity and mortality(2,3). Promoting the uptake of COVID-19 vaccines requires people's willingness to get vaccinated and ensuring proper communication of information through trusted sources to aid vaccine acceptance (4,5) .

Ethiopia started the COVID-19 vaccination campaign in March 2021 with the Astra Zeneca vaccine, manufactured by the Serum Institute of India and supplied by the COVID-19 Vaccines Global Access (COVAX) initiative (6). The Ministry of Health launched a national ceremony to initiate the COVID-19 vaccine rollout in the presence of different stakeholders, including religious representatives. Initially, healthcare providers and older people with comorbidities were prioritized and given two doses of AstraZeneca vaccines (6,7) . In the later stage, the Johnson & Johnson vaccine, Sinopharm, and Pfizer vaccines were included in the campaign as they became available (7,8) .

Vaccines need to be effectively utilized by the target population to bring about the intended effect of controlling the pandemic. However, despite the availability of vaccines and vaccination services, vaccine hesitancy- refusal or delay to accept a vaccine, has become a significant concern (9,10) . WHO considers vaccine hesitancy as one of the top 10 Global Health threats (11). Concerns about vaccine safety, efficacy, and side effects have driven vaccine hesitancy related to COVID-19 worldwide (12,13) . Moreover, the slow rollout and politicization of vaccines in Africa have fueled rumors, doubts, and conspiracy theories about these vaccines (14,15). Lack of trust in the government and the private sector, reading misinformation on social media, and the history of vaccination programs in a country have been further reasons for vaccine hesitancy among health workers and the population in Africa (13,14,16,17) . Studies have shown high COVID-19 vaccine hesitancy among the Ethiopian population and some factors mentioned for the hesitancy were having social media as a primary information source, fear of side effects of the vaccine and thinking it as a biological weapon and doubts about the vaccine (15,18–20).

Global surveys show that religious factors are the third most commonly reported reason for vaccine hesitancy globally (21) . Religious views can affect how people react to vaccinations, causing reactions like vaccine hesitancy despite conveying medically sound and scientifically proven

For peer review only

For peer review only - http://bmjopen.bmj.com/site/about/guidelines.xhtml

information/evidence (22) . In the context of the COVID-19 pandemic, concerns have been raised that religious communities may serve as the basis for misinformation and unfounded theories that undermine the use of COVID-19 vaccines (23). Thus, the importance of faith-leaders in addressing vaccine hesitancy has received increasing attention in global health research (24) .

Ethiopia is a religious country where more than 97% religious population, and faith-leaders hold significant societal positions (25) . It has been well documented that faith-leaders are embedded in society with high-influencing positions and often have considerable leverage with state and non-state powers due to the size of their constituencies (26–29). Therefore, engagement with faith-leaders is critical as they are known to be gatekeepers to local communities, with considerable influence on their communities' beliefs and behavior. Thus, this study aimed to explore faith leaders' perspectives on the COVID-19 vaccine and the role faith-leaders can play in building COVID-19 vaccine trust.

Methods

Study setting

This study was conducted in Addis Ababa, the capital city of Ethiopia. Orthodox Christianity, Islam, and Protestantism are the main religions in Ethiopia (25) . The Inter-Religious Council of Ethiopia (IRCE) was established in 2010 to promote inter-religious harmony in Ethiopia (30). IRCE is represented at federal, regional, and district levels. The IRCE head office is located in Addis Ababa. This council comprises seven religious groups, each with a representative in the head office.

Study design and aim

A qualitative study design using in-depth interviews was used to explore faith leaders' perceptions of the COVID-19 vaccine and the role faith-leaders can play in building COVID-19 vaccine trust.

Study population

The participants of this study are faith-leaders selected from the seven religious groups represented in the IRCE, which comprises Ethiopian Orthodox Tewahido Church, Islam, 7th Day Adventist, Evangelical churches fellowship of Ethiopia, Ethiopian Kale Heywet church, Ethiopian Evangelical Church Mekane Eyesus, and Ethiopian Catholic church.

For peer review only - http://bmjopen.bmj.com/site/about/guidelines.xhtml

Study sample and sampling procedure

Purposive sampling was used to identify appropriate study participants. The purpose of reaching out to the inter-religious council was with the notion that all the country's major religions were represented, and interviewing these participants would provide us with a comprehensive understanding of the country's context. We identified the seven religious entities constituting the IRCE through the inter-religious council. From these seven religions, informative individuals were selected in consultation with the head office representatives of each religion. We selected three study respondents from each religious entity: two faith-leaders at the head offices and one from the religious institutions (Churches and Mosques), culminating in 21 participants. The interviews conducted later in the study did not generate new information, so we assumed reaching data saturation.

Data collection procedures

In-depth interviews (IDI) were used for data collection. The themes of the interview guide were developed based on our background research. Themes included faith-leaders understanding COVID-19, rumors and uncertainty around the COVID-19 vaccine, and the role of faith-leaders in controlling the pandemic. The research team developed the interview guide in English and translated it into Amharic (the official language). Two research assistants with extensive experience in qualitative data collection were recruited to collect the data. The research assistants were trained for two days on the interview guides, the purpose of the study, and research ethics. Verbal consent was obtained before the interview. All interviews were audio recorded, and additional notes were taken during the in-depth interview. The IDIs were conducted in a private and quiet place, mainly in an area preferred by the participants, their offices most of the time. The interviews took 40 - 90 minutes and were conducted on dates and times convenient to the participants. COVID-19 prevention methods such as keeping physical distance, using hand sanitizers, and wearing masks were strictly implemented during the data gathering.

Data analysis

The research assistants transcribed all interviews verbatim and then translated from Amharic to English. Transcripts and translations were checked for accuracy and consistency by the two independent persons in the research group who did not conduct the interviews. Field notes taken during the IDIs were integrated into the transcription. KY, FW, and SHvW conducted a thematic

For peer review only - http://bmjopen.bmj.com/site/about/guidelines.xhtml

qualitative analysis following Braun and Clarke's method (31). The researchers developed an intensive codebook and kept all audit trail records, such as data reduction notes, during the analysis. The codebooks included definitions and example responses. Regular meetings, both in person and virtually, were made among the researchers to ensure consistency and validity. The Thematic analysis was done using Atlas.ti software version 7.5.16. Working with multiple coders aided in minimizing the subjective bias of researchers. The coding process was continued until the data was exhausted. Then, the codes were categorized, and themes were created. The themes were developed and refined following several team meetings.

Patient and public involvement

No patient is involved.

Ethical considerations

Participation in this study was entirely voluntary, and informed consent was obtained from all participants. The study procedures, benefits, anticipated risks or discomforts of participating, and right to withdraw were explained. Principles of anonymity and confidentiality were applied to protect the participants' identities by assigning codes to them. The audio recordings were destroyed after the completion and verification of the transcription and translations. The study was conducted following national ethical standards and the declaration of Helsinki (32).

For peer review only

For peer review only - http://bmjopen.bmj.com/site/about/guidelines.xhtml

Results

The thematic analysis revealed three themes and 15 categories, Table 1. A category was created when many codes or data extracts described that specific finding. We only use one data extract to describe the category when presenting the data in this section.

Table 1 Themes and defined categories based on thematic analysis.

Theme	Category
Faith-leaders were aware of the risks of the COVID-19 pandemic, and they and their congregations were seriously affected by the pandemic.	Faith-leaders were aware of the risks of the COVID-19 pandemic.
	Solitude in difficult times
	Financial loss for faith institutions
	Vulnerable groups are left behind.
	Spiritual crisis
Faith-leaders were important allies during a pandemic	Faith-leaders were trusted
	Faith-leaders collaborated with government and health professionals.
	Faith-leaders engaged in public health prevention using spiritual reasoning.
	Faith-leaders trusted medical science.
	Faith-leaders were actively engaged in COVID-19 prevention methods.
Faith-leaders were intrigued by rumors and uncertainty around COVID-19 vaccine	Faith-leaders trusted vaccines
	Rumors about vaccines were rampant in the community.
	Spiritual rumors: 666
	Faith-leaders tried to address concerns.
	Faith-leaders were unsure how to respond to Rumors.

Faith-leaders were aware of the COVID-19, and they and their congregations had been seriously affected by the pandemic.

Although most faith-leaders in this study ascribed a spiritual meaning to the advent of the COVID-19 pandemic, all faith-leaders interviewed were aware of the risk and severity of the COVID-19 pandemic. They were well-informed and described in great detail the severe effects it had on their religious practice and the members of their spiritual community. In the words of a faith leader:

For peer review only - http://bmjopen.bmj.com/site/about/guidelines.xhtml

"There is nothing it did not touch; it has affected our social life, economy, religious relations, our office, our work... We have lost so many people." (IDI-12)

Faith-leaders described the implications of solitude under challenging times, particularly during times of mourning and grief. Participants expressed profound sadness that they were unable to console individuals and support them during times of grief: *"We could not even comfort the families of the deceased after the funeral. We consider spiritual support as a psychosocial treatment, but we failed to offer that too."* (IDI-4)

Moreover, faith-leaders described a spiritual crisis in their institutions whereby relationships between faith-leaders and those attending religious institutions had been harmed. This is mainly due to not allowing people to participate in religious services, which led to conflicts in several religious institutions. Faith-leaders described a change in spiritual practice and organization, which has remained in practice:

> *It* [COVID-19] *even created another culture; older people and people with some medical conditions became too scared to come to our congregation even after we were allowed to gather again. In our religion, Christianity, the New Testament encourages and teaches togetherness to grow in holiness. So, it affected that core value.* (IDI-6)

Faith-leaders further described the financial implications for their institutions. Due to the loss of participants in religious services, income diminished substantially. Businesses surrounding religious institutions were further negatively affected. This led to significant staff cuts at religious institutions. Moreover, it led to the closure of social activities and programs used to serve the needs of the poorest: *"With the limited money we got, we were only able to pay salaries church ministers; there was nothing left to give to those who are in need."* (IDI-4)

Despite these challenges, faith-leaders described innovative approaches to countering challenges. This includes the use of social media to reach the community:

> *We tried to solve this and reach our members through technology by calling them over the phone and using the Zoom application. Fortunately, we launched a Television program a month or two before the pandemic called Hope Channel, and we used that to preach and praise the Lord...* (IDI-14)

Active fundraising in the community was further organized to make up for the financial loss:

For peer review only

For peer review only - http://bmjopen.bmj.com/site/about/guidelines.xhtml

When everyone was told to stay home, it was hard for our community because they had no money or food to eat, especially during fasting. So, we have tried our best to provide them food for the fasting period. We did that here in Addis Ababa and in some other areas, too. For that, we have collected over 30 million birr from investors and distributed it to others. (IDI-16)

Faith-leaders are important allies during a pandemic.

The second theme summarizes faith-leaders and their institutions' work during the pandemic. To begin with, most faith-leaders collaborated closely with the Ministry of Health and health professionals to inform their congregations about the disease and preventive measures. The collaboration between the government and religious institutions appears to have been effective, and faith-leaders trusted the information and directives of the Ministry of Health:

"In our church, I invited different medical doctors to convey health messages about getting vaccinated and safety measures practices recommended by the health ministry and the government. Based on these messages, I encourage people to practice what they have heard." (IDI-20)

In turn, faith-leaders consider themselves highly trusted within their communities and congregations. They are consulted in times of hardship and on important family matters.

"Religious leaders have many roles not only in controlling pandemics but also in other national affairs. This is because many people trust religious leaders; they obey them. Their word is associated with God, and what they say will get higher acceptance. Instead of a soldier speaking with a weapon, a priest holding a cross is more heard and accepted. This is a well-known fact in our country" (IDI-2).

They were also consulted about questions regarding the COVID-19 pandemic, mainly rumors about the origin of the pandemic and vaccines. There are numerous examples of how faith-leaders describe following directives, implementing recommended interventions (hand washing, mask use, social distancing), and actively informing the community about importance of the COVID-19 vaccine.

Questions are forwarded to me; we often convey a message when we finish teaching in the church. That people should follow the instructions. In general, we

For peer review only - http://bmjopen.bmj.com/site/about/guidelines.xhtml

> *tell them God loves us as we obey, wash our hands, we should respect each other, and the vaccine is also important you should take it. What we do here is critical; we are spiritual doctors.* (IDI-3)

Faith-leaders saw themselves as key in implementing measures to address the pandemic and proactively raising awareness to help implement directives.

> *We intensively created awareness, stressing that the vaccine is one of the major remedies for this disease. We assigned responsible persons and Imams who can transmit these messages during the five prayer times in every Mosque. Regarding awareness creation, we successfully changed people's understanding from wrong to right direction.* (IDI-16)

Faith-leaders described how they used religious scriptures to promote guidelines to reduce transmission. This was particularly important when the community challenged them regarding these measures.

> *The Bible supports all the recommended preventive safety measures for COVID-19. So, if parishioners come with such concerns, I will teach them this according to the Bible and guide them in the right direction.* (IDI-2)

Several faith leaders, regardless of the denomination, described examples of how they used spiritual reasoning to address concerns: *…the Bible says that Luke, the Evangelist, was one of the writers of the Bible who had medical knowledge. Therefore, the doctrine of the Church does not contradict such wisdom.* (IDI-11) Almost all faith-leaders in this study described a strong trust in science and use religious reasoning to support this. In the words of a faith leader*: "God protects us from death through faith and science"* (IDI-21)

Faith-leaders are faced with rumors and uncertainty around the COVID-19 vaccine.

A significant challenge described by faith-leaders was the rumors about the COVID-19 vaccine they encountered. This section summarizes faith leaders' different engagements with these rumors. Despite the strong commitment of faith-leaders to implement preventative measures, they faced challenges in addressing rumors about vaccines. Most, but not all, faith-leaders in this study trust the COVID-19 vaccine. They described their trust in science and the government and used religious reasoning to support their trust. Many faith-leaders were publicly vaccinated to build trust in the vaccine: *"I and other faith-leaders of different religions in Addis Ababa got vaccinated in*

For peer review only - http://bmjopen.bmj.com/site/about/guidelines.xhtml

public; we did that to be role models to our parishioners." (IDI-13)

Participants described numerous and consistent rumors encountered in the community. The COVID-19 vaccine was considered dangerous because it was sent from abroad and was therefore not trusted: *"Now Ethiopia is in disagreement with America as we all know, what if they send this vaccine to destroy us and attack us?"* (IDI-14). There were rumors that the COVID-19 vaccine represented 666 – the mark of the beast.

> *There is a religious teaching about 666*[1]*. I guess you have heard about that in every church. So, they say this vaccine is used to insert satanic 666 into our bodies. So, they say Satan makes the vaccine, so this is satanic teaching. They say participating in this is like partnering with Satan.* (IDI-13)

Moreover, a repeated concern that faith-leaders faced, and some addressed themselves, was the effect of the COVID-19 vaccine on fertility. Faith-leaders described how rumors affected their own beliefs, and they described challenges in addressing these questions:

> *..it is rumored that getting vaccinated can cause infertility. They also say that developed countries fabricate this disease to minimize the population of highly populated countries like African countries. There were so many questions. It was tough to answer such questions.* (IDI-16)

Few faith-leaders actively countered the rumors, but those who did use spiritual reasoning to do so. For example:

> *When they say it will make you infertile, I will say, are you a prophet? Have you been given the gift of prophesying? So this shows an awareness problem due to a lack of awareness; that's how I see it.* (IDI-3)

However, several faith-leaders described that they got uncertain about the vaccine as a result of the rumors: "*It's best to try to understand what they want to say. I don't have evidence it might be what they said or it may not. So the main thing is believing."* (IDI-21)

Moreover, some described that they could not address those concerns. They were more comfortable preaching about all other public health prevention measures than the vaccine.

> *One of our [faith-leaders] tasks is to educate the community on preventing these outbreaks as health professionals recommended. Teaching about the*

[1] *According to most manuscripts of the New Testament and in English translations of the Bible, the number of the beast is six hundred sixty-six. (Book of Revelation 13:15-18, Wikipedia)*

For peer review only - http://bmjopen.bmj.com/site/about/guidelines.xhtml

disease/ pandemic is comfortable, but I cannot say anything about specific vaccines. (IDI-11)

For peer review only

For peer review only - http://bmjopen.bmj.com/site/about/guidelines.xhtml

Discussion

This qualitative study explored faith leaders' role in combating the pandemic and in building COVID-19 vaccine trust in Addis Ababa. The findings of our study showed that most faith-leaders included in this study clearly understand the risks associated with COVID-19. Due to the advent of the COVID-19 pandemic, faith-leaders and their congregations have faced solitude in challenging times. Additionally, religious institutions have encountered significant financial and spiritual crises. Our study also revealed that faith-leaders were actively engaged in pandemic measures and held strong beliefs in the medical sciences, including vaccines. However, faith-leaders struggled to address COVID-19 vaccine rumors in their communities despite the high trust in vaccines and medical science.

This study indicated that faith-leaders were well-informed about the risks of COVID-19. This could be due to reasonable access to sources regarding the COVID-19 pandemic due to their networks, mass media, and social media exposure. Our findings disclosed that due to the COVID-19 pandemic, vulnerable groups, faith leaders, and their congregations have faced solitude during challenging times and significant financial and spiritual crises. This finding aligns with a qualitative study conducted in Ghana, which showed that the COVID-19 pandemic caused economic challenges to the congregants, a decline in spiritual life, and a loss of fellowship and community (33). In another qualitative study conducted on United Methodist pastors in one of the two Annual Conferences in North Carolina, Pastors reported that COVID-19 fundamentally unsettled the routine works of the ministry (34).

This study also identified innovative approaches faith-leaders use to tackle the challenges. This includes using social media to reach the community and fundraising to compensate for the financial loss. A qualitative study on Ghanaian Christian Leaders also showed that as a response to the ban, most participants in their research explicitly reported that they moved at least some activities online, which included conventional activities, such as posting prayer topics and live streaming of services for audiences who wished to view them synchronously(35). Similarly, religious authorities in Uganda played a critical role in delivering public health messages on COVID-19 risk communication using the web .

According to a recent systematic review that assessed religious communities' role, religion has acted as an essential platform for inter-sectoral collaboration with science and government to

For peer review only - http://bmjopen.bmj.com/site/about/guidelines.xhtml

combat COVID-19(36) . National, regional, and local faith-leaders have high levels of influence and community-organizing capabilities. Faith-leaders are effective messengers endorsing vaccination compared to other potential messengers(28) . They can help frame approaches that make them more likely to be accepted in their communities. Governments should build trust with faith-leaders and integrate them into planning, decision-making, and implementation at every level of their COVID-19 response (37). The findings in this study also showed that there was a strong collaboration between the government and religious institutions, which appeared to have been effective, and faith-leaders trusted the information and directives of the Ministry of Health. This might be due to the closeness of the religious institutions to each other and the government through a well-founded and organized inter-religious council that collaborates with the government on different national agendas (30) . Besides, all faith-leaders interviewed for this study consider themselves highly trusted within their communities and congregations. They were consulted about questions regarding the COVID-19 pandemic, mainly rumors about the origin of the pandemic and vaccines. Due to that, most of the study participants saw themselves as critical actors in implementing the safety measures and in proactively raising awareness to help implement directives to address the pandemic. Since faith-leaders are often highly influential community leaders who are 'listened to by the community members, they often underutilize the potential for catalyzing change (24) . In line with this finding, a qualitative case study conducted in Indonesia showed that the faith-leaders supported the health directives designed to reduce high transmission risk(38) . All the scholars and faith-leaders stated that funerals, according to the health protocols issued by the authorities, should be conducted considering potential disease transmission. Some Islamic organizations have developed their guidelines for COVID-19 by modifying the religious values of certain institutions to prevent the spread of infectious diseases (38) .

This study revealed that regardless of the denomination, almost all faith-leaders who participated in this study said they trust in science and use religious scriptures and spiritual reasoning to promote guidelines to reduce the transmission of COVID-19. A study has also shown that many faith-leaders see the vaccine as a message of hope. Pope Francis also indicated everyone's moral obligation to be vaccinated (40). Our study participants added that many faith-leaders were publicly vaccinated to build trust in the vaccine. This was particularly important when the community challenged them regarding these measures. In agreement with that, findings of a qualitative study conducted in Leeds, United Kingdom, also revealed that faith-leaders could play

For peer review only - http://bmjopen.bmj.com/site/about/guidelines.xhtml

a vital role in the health behavior of their congregants (27) . Faith-leaders can influence health behavior not only on the individual but also on a sociocultural and environmental level. They exert such influence through several mediators, including as scriptural power, social influencer, and serving as role models (27) . Our results showed most of the faith-leaders faced numerous and consistent rumors in the community. They were affected by the rumors they heard about the COVID-19 vaccine and faced challenges in addressing such questions.

The African Center for Strategic Studies reported that the preponderance of COVID-19 vaccine myths is causing many Africans to forego vaccinations when new, more transmissible coronavirus variants spread across the continent (39) . The report indicated several myths, including live viruses are injected to cause death after vaccination; the vaccine causes infertility by altering the DNA to reduce Africa's population, and other serious side effects that are worse than contracting the virus, and some claim that vaccines are a cover to implant traceable microchips (39) . Such myths and similar rumors may lead to COVID-19 vaccine hesitancy in the general community. In line with these findings, a population-based online survey conducted in Ethiopia showed that only 31.4% of participants were willing to receive the COVID-19 vaccine (19). One of the strategies to counter vaccine hesitancy is to follow a multisectoral approach that involves the collaboration between various stakeholders, such as government, private companies, religious groups, and other agencies, to leverage the knowledge, expertise, and resources, thereby enabling the creation of longstanding public trust of vaccines (27,40) . Given the importance of faith-leaders and their trust in science, equipping faith-leaders with the knowledge and skills to address rumors appears indispensable.

Limitations of the study can lay in the fact that we only interviewed faith-leaders included in the structured inter-religious council and did not directly interview those outside of this structure to capture their perception and role. Although the findings cannot be generalized, inferential generalizations can be drawn, and results may be relevant for other similar settings.

Conclusion

Faith-leaders have played an essential role in mitigating the COVID-19 pandemic in Ethiopia. Faith-leaders have followed and implemented government directives and promoted the vaccine, further amplifying their important alliance in public health. However, they have been challenged with different rumors about the vaccine, which they struggled to address as they were not equipped

For peer review only - http://bmjopen.bmj.com/site/about/guidelines.xhtml

to do so. Thus, empowering faith-leaders to address rumors about new vaccines could have significant public health implications for ongoing and future pandemics.

Contributor statement – YB, SHvW, FW, and KY designed the study. HM & HW conducted the interviews, transcriptions, and translations. KY, FW, HM, WT, and SHvW were involved in the coding and conducted the analysis. All authors collaborated on the writing of the manuscript, and the final draft was written by KY & FW. All authors have read and approved the final manuscript.

Competing Interests - No conflicts of interest to declare.

Funding – The Addis Continental Institute of Public Health, Ethiopia, funded the study. SHvW is the recipient of a Postdoc grant awardee by FORTE (STY396 2021/0001) in Sweden.

Data sharing statement – Data are available upon reasonable request

Ethics approval- Ethical clearance was obtained from the Institutional Review Board (IRB) of Addis Continental Institute of Public Health (ACIPH/IRB/010/2021).

Acknowledgments – We would like to acknowledge the inter-religious council of Ethiopia for their collaboration for the study.

eview only

For peer review only - http://bmjopen.bmj.com/site/about/guidelines.xhtml

Reference

1. Legge D, Kim S. Equitable Access to COVID-19 Vaccines: Cooperation around Research and Production Capacity Is Critical. 2021 Jul 27; Available from: https://opal.latrobe.edu.au/articles/journal_contribution/Equitable_Access_to_COVID-19_Vaccines_Cooperation_around_Research_and_Production_Capacity_Is_Critical/15050310/1

2. Bardosh K, Figueiredo A de, Gur-Arie R, Jamrozik E, Doidge J, Lemmens T, et al. The unintended consequences of COVID-19 vaccine policy: why mandates, passports and restrictions may cause more harm than good. BMJ Glob Health. 2022 May 1;7(5):e008684.

3. Pilkington V, Keestra SM, Hill A. Global COVID-19 Vaccine Inequity: Failures in the First Year of Distribution and Potential Solutions for the Future. Front Public Health. 2022 Mar 7;10:821117.

4. World Bank. Accelerating COVID-19 Vaccine Deployment : Removing Obstacles to Boost Coverage Levels and Protect Those at High-risk (English). Available from: https://documents.worldbank.org/en/publication/documents-reports/documentdetail

5. Druedahl LC, Minssen T, Price WN. Collaboration in times of crisis: A study on COVID-19 vaccine R&D partnerships. Vaccine. 2021 Oct 8;39(42):6291–5.

6. WHO | Regional Office for Africa. 2023. Ethiopia introduces COVID-19 vaccine in a national launching ceremony. Available from: https://www.afro.who.int/news/ethiopia-introduces-covid-19-vaccine-national-launching-ceremony

7. COVAX | MINISTRY OF HEALTH - Ethiopia. Available from: https://www.moh.gov.et/site/covax

8. The Ministry of Health has received 10 million doses of covid 19 vaccine from the Chinese government. | MINISTRY OF HEALTH - Ethiopia . Available from: https://www.moh.gov.et/site/node/352

9. Mesch GS, Schwirian KP. Vaccination hesitancy: fear, trust, and exposure expectancy of an Ebola outbreak. Heliyon. 2019 Jul 18;5(7):e02016.

10. Sallam M. COVID-19 Vaccine Hesitancy Worldwide: A Concise Systematic Review of Vaccine Acceptance Rates. Vaccines. 2021 Feb 16;9(2):160.

11. Scheres J, Kuszewski K. The Ten Threats to Global Health in 2018 and 2019. A welcome and informative communication of WHO to everybody. Zdr Publiczne Zarządzanie. 2019 Jan 1;17:2–8.

12. Solís Arce JS, Warren SS, Meriggi NF, Scacco A, McMurry N, Voors M, et al. COVID-19 vaccine acceptance and hesitancy in low- and middle-income countries. Nat Med. 2021;27(8):1385–94.

13. Biswas MdR, Alzubaidi MS, Shah U, Abd-Alrazaq AA, Shah Z. A Scoping Review to Find Out Worldwide COVID-19 Vaccine Hesitancy and Its Underlying Determinants. Vaccines. 2021 Oct 25;9(11):1243.

14. Ekwebelem OC, Onyeaka H, Yunusa I, Miri T, Onwuneme YM, Eunice AT, et al. Do we trust the government? Attributes of COVID-19 vaccine hesitancy and acceptance in Nigeria. AIMS Med Sci. 2022;9(2):268–82.

For peer review only - http://bmjopen.bmj.com/site/about/guidelines.xhtml

15. Dereje N, Tesfaye A, Tamene B, Alemeshet D, Abe H, Tesfa N, et al. COVID-19 vaccine hesitancy in Addis Ababa, Ethiopia: a mixed-method study. BMJ Open. 2022 May;12(5):e052432.

16. Ekwebelem OC, Nnorom-Dike OV, Aborode AT, Ekwebelem NC, Aleke JC, Ofielu ES. Eradication of wild poliovirus in Nigeria: Lessons learnt. Public Health Pract. 2021 May 15;2:100144.

17. Jegede AS. What Led to the Nigerian Boycott of the Polio Vaccination Campaign? PLOS Med. 2007 Mar 20;4(3):e73.

18. Terefa DR, Shama AT, Feyisa BR, Desisa AE, Geta ET, Cheme MC, et al. COVID-19 Vaccine Uptake and Associated Factors Among Health Professionals in Ethiopia. Infect Drug Resist. 2021;14:5531.

19. Belsti Y, Gela YY, Akalu Y, Dagnew B, Getnet M, Abdu Seid M, et al. Willingness of Ethiopian Population to Receive COVID-19 Vaccine. J Multidiscip Healthc. 2021;14:1233–43.

20. Mohammed R, Nguse TM, Habte BM, Fentie AM, Gebretekle GB. COVID-19 vaccine hesitancy among Ethiopian healthcare workers. PLOS ONE. 2021 Dec 17;16(12):e0261125.

21. USAID MOMENTUM. Effects of Faith Actor Engagement on the Uptake and Coverage of Immunization in Low- and Middle-Income Countries. Available from: https://usaidmomentum.org/resource/vaccine-hesitancy-report/

22. Garcia LL, Yap JFC. The role of religiosity in COVID-19 vaccine hesitancy. J Public Health Oxf Engl. 2021 Jun 3;fdab192.

23. Lee Rogers R, Powe N. COVID-19 Information Sources and Misinformation by Faith Community. Inq J Med Care Organ Provis Financ. 2022 May 30;59:00469580221081388.

24. Bartelink B, le Roux E, Palm S. Sleeping Giants: Mobilizing faith leaders as agents of change. London: Tearfund; 2017.

25. The DHS Program - Ethiopia: DHS, 2016 - Final Report (English). Available from: https://dhsprogram.com/publications/publication-fr328-dhs-final-reports.cfm

26. Anshel M, Smith M. The Role of Religious Leaders in Promoting Healthy Habits in Religious Institutions. J Relig Health. 2013 Mar 21;53.

27. Heward-Mills NL, Atuhaire C, Spoors C, Pemunta NV, Priebe G, Cumber SN. The role of faith leaders in influencing health behaviour: a qualitative exploration on the views of Black African Christians in Leeds, United Kingdom. Pan Afr Med J. 2018;30:199.

28. Viskupic F, Wiltse D. The Messenger Matters: Religious Leaders and Overcoming COVID-19 Vaccine Hesitancy. 2021.

29. Syed U, Kapera O, Chandrasekhar A, Baylor BT, Hassan A, Magalhães M, et al. The Role of Faith-Based Organizations in Improving Vaccination Confidence & Addressing Vaccination Disparities to Help Improve Vaccine Uptake: A Systematic Review. Vaccines. 2023 Feb;11(2):449.

30. Inter-Religious Council of Ethiopia. 2013. Available from: https://www.uri.org/who-we-are/cooperation-circle/inter-religious-council-ethiopia

For peer review only

For peer review only - http://bmjopen.bmj.com/site/about/guidelines.xhtml

31. Braun V, Clarke V. Using thematic analysis in psychology. Qual Res Psychol. 2006 Jan 1;3:77–101.

32. World Medical Association. World Medical Association Declaration of Helsinki: Ethical Principles for Medical Research Involving Human Subjects. JAMA. 2013 Nov 27;310(20):2191–4.

33. Osei-Kuffour F, Kwasi Peprah W, Marfo Sarfo D, Osei Yeboah B. COVID-19 Impact on Church Cash Inflows in Ghana as Moderated by Location Profile. Appl Finance Account. 2022 Oct 13;8(1):6.

34. Johnston EF, Eagle DE, Headley J, Holleman A. Pastoral Ministry in Unsettled Times: A Qualitative Study of the Experiences of Clergy During the COVID-19 Pandemic. Rev Relig Res. 2022;64(2):375–97.

35. Adams G, Osei-Tutu A, Affram AA, Phillips-Kumaga L, Dzokoto VAA. Implications of COVID-19 Innovations for Social Interaction: Provisional Insights From a Qualitative Study of Ghanaian Christian Leaders. Front Psychol. 2022 May 24;13:647979.

36. Lee M, Lim H, Xavier MS, Lee EY. "A Divine Infection": A Systematic Review on the Roles of Religious Communities During the Early Stage of COVID-19. J Relig Health. 2022;61(1):866–919.

37. Tony-Blair-Institute-Working-With-Religious-Leaders-to-Support-Public-Health-Measures.pdf [Internet]. [cited 2023 Dec 1]. Available from: https://docs.google.com/viewerng/viewer?url=https://jliflc.com/wp-content/uploads/2020/05/Tony-Blair-Institute-Working-With-Religious-Leaders-to-Support-Public-Health-Measures.pdf

38. Nurhayati N, Purnama TB. Funeral Processes During the COVID-19 Pandemic: Perceptions Among Islamic Religious Leaders in Indonesia. J Relig Health. 2021;60(5):3418–33.

39. Studies the AC for S. Dispelling COVID Vaccine Myths in Africa. Africa Center for Strategic Studies. Available from: https://africacenter.org/spotlight/dispelling-covid-vaccine-myths-in-africa/

40. Galang JRF. Science and religion for COVID-19 vaccine promotion. J Public Health Oxf Engl. 2021 Sep 22;43(3):e513–4.

For peer review only - http://bmjopen.bmj.com/site/about/guidelines.xhtml

For peer review only

Consolidated criteria for reporting qualitative studies (COREQ): 32-item checklist

Please indicate in which section each item has been reported in your manuscript. If you do not feel an item applies to your manuscript, please enter N/A.

For further information about the COREQ guidelines, please see Tong *et al.*, 2017: https://doi.org/10.1093/intqhc/mzm042

No.	Item	Description	Page #
**Domain 1: Research team and reflexivity**			
Personal characteristics			
1.	Interviewer/facilitator	Which author/s conducted the interview or focus group?	15
2.	Credentials	What were the researcher's credentials? *E.g. PhD, MD*	PhD, MPH, MD
3.	Occupation	What was their occupation at the time of the study?	Professor, Post doctoral researcher, Assistant Professors, Lecturer and research associate
4.	Gender	Was the researcher male or female?	Kalkidan, Firehiwot, Workagnehu, Hanna are females whereas Yemane and Habtamu are males
5.	Experience and training	What experience or training did the researcher have?	Minimum of 5 years and above experience on research and training
Relationship with participants			
6.	Relationship established	Was a relationship established prior to study commencement?	N/A
7.	Participant knowledgeof the interviewer	What did the participants know about the researcher? *E.g. Personal goals, reasons for doing the research*	N/A
8.	Interviewer characteristics	What characteristics were reported about the interviewer/facilitator? *E.g. Bias, assumptions, reasons and interests in the research topic*	N/A
**Domain 2: Study design**			
Theoretical framework			
9.	Methodological orientation and theory	What methodological orientation was stated to underpin the study? *E.g. grounded theory, discourse analysis, ethnography, phenomenology, content analysis*	5
Participant selection			
10.	Sampling	How were participants selected? *E.g. purposive, convenience, consecutive, snowball*	5
11.	Method of approach	How were participants approached? *E.g. face-to-face, telephone, mail, email*	5
12.	Sample size	How many participants were in the study?	5
13.	Non-participation	How many people refused to participate or dropped out? What were the reasons for this?	N/A

For peer review only

Setting			
14.	Setting of data collection	Where was the data collected? *E.g. home, clinic, workplace*	5
15.	Presence of non-participants	Was anyone else present besides the participants and researchers?	5

For peer review only

16.	Description of sample	What are the important characteristics of the sample? *E.g. demographic data, date*	5
Data collection			
17.	Interview guide	Were questions, prompts, guides provided by the authors? Was it pilot tested?	5
18.	Repeat interviews	Were repeat interviews carried out? If yes, how many?	N/A
19.	Audio/visual recording	Did the research use audio or visual recording to collect the data?	5
20.	Field notes	Were field notes made during and/or after the interview or focus group?	5
21.	Duration	What was the duration of the interviews or focus group?	5
22.	Data saturation	Was data saturation discussed?	No
23.	Transcripts returned	Were transcripts returned to participants for comment and/or correction?	N/A
**Domain 3: analysis and findings**			
Data analysis			
24.	Number of data coders	How many data coders coded the data?	15
25.	Description of the coding tree	Did authors provide a description of the coding tree?	5 & 7
26.	Derivation of themes	Were themes identified in advance or derived from the data?	5
27.	Software	What software, if applicable, was used to manage the data?	5
28.	Participant checking	Did participants provide feedback on the findings?	N/A
Reporting			
29.	Quotations presented	Were participant quotations presented toillustrate the themes / findings? Was each quotation identified? *E.g. Participant number*	8,9,10 & 11
30.	Data and findings consistent	Was there consistency between the data presented and the findings?	12, 13 & 14
31.	Clarity of major themes	Were major themes clearly presented in the findings?	7
32.	Clarity of minor themes	Is there a description of diverse cases or discussion of minor themes?	N/A

When submitting your manuscript via the online submission form, please upload the completed checklist as a Figure/supplementary file.

If you would like this checklist to be included alongside your article, we ask that you upload the completed checklist to an online repository and include the guideline type, name of the repository, DOI and license in the *Data availability* section of your manuscript.

Developed from: Allison Tong, Peter Sainsbury, Jonathan Craig, Consolidated criteria for reporting qualitative research (COREQ): a 32-item checklist for interviews and focus groups, International Journal for Quality in Health Care, Volume 19, Issue 6, December 2007, Pages 349–357, https://doi.org/10.1093/intqhc/mzm042

For peer review only

BMJ Open

"God protects us from death through faith and science": A qualitative study on the role of the faith-leaders in combating the COVID-19 pandemic and in building COVID-19 vaccine trust in Addis Ababa, Ethiopia.

Journal:	*BMJ Open*
Manuscript ID	bmjopen-2023-071566.R2
Article Type:	Original research
Date Submitted by the Author:	12-Feb-2024

Complete List of Authors:	Yibeltal, Kalkidan; Addis Continental Institute of Public Health, Department of Reproductive health and Population<br>Workneh, Firehiwot ; Addis Continental Institute of Public Health, Epidemiology and Biostatistics<br>Melesse, Hanna; Addis Continental Institute of Public Health, Department of Epidemiology and Biostatistics<br>Wolde, Habtamu; Independent Consultant<br>Kidane, Workagegnehu Tarekegn; Addis Continental Institute of Public Health, Department of Nutrition and Behavioral Sciences<br>Berhane, Yemane ; Addis Continental Institute of Public Health, Department of Epidemiology and Biostatistics<br>Herzig van Wees, Sibylle; Karolinska Institute, Department of Global Public Health

**Primary Subject Heading**:	Public health
Secondary Subject Heading:	Global health
Keywords:	COVID-19, Public health < INFECTIOUS DISEASES, QUALITATIVE RESEARCH

SCHOLARONE™ Manuscripts

I, the Submitting Author has the right to grant and does grant on behalf of all authors of the Work (as defined in the below author licence), an exclusive licence and/or a non-exclusive licence for contributions from authors who are: i) UK Crown employees; ii) where BMJ has agreed a CC-BY licence shall apply, and/or iii) in accordance with the terms applicable for US Federal Government officers or employees acting as part of their official duties; on a worldwide, perpetual, irrevocable, royalty-free basis to BMJ Publishing Group Ltd ("BMJ") its licensees and where the relevant Journal is co-owned by BMJ to the co-owners of the Journal, to publish the Work in this journal and any other BMJ products and to exploit all rights, as set out in our licence.

The Submitting Author accepts and understands that any supply made under these terms is made by BMJ to the Submitting Author unless you are acting as an employee on behalf of your employer or a postgraduate student of an affiliated institution which is paying any applicable article publishing charge ("APC") for Open Access articles. Where the Submitting Author wishes to make the Work available on an Open Access basis (and intends to pay the relevant APC), the terms of reuse of such Open Access shall be governed by a Creative Commons licence – details of these licences and which Creative Commons licence will apply to this Work are set out in our licence referred to above.

Other than as permitted in any relevant BMJ Author's Self Archiving Policies, I confirm this Work has not been accepted for publication elsewhere, is not being considered for publication elsewhere and does not duplicate material already published. I confirm all authors consent to publication of this Work and authorise the granting of this licence.

For peer review only

"God protects us from death through faith and science": A qualitative study on the role of the faith-leaders in combating the COVID-19 pandemic and in building COVID-19 vaccine trust in Addis Ababa, Ethiopia.

Co-authors

Kalkidan Yibeltal - Department Reproductive Health and Population. Addis Continental Institute of Public Health

Firehiwot Workneh - Department of Epidemiology and Biostatistics. Addis Continental Institute of Public Health, Addis Ababa, Ethiopia

Hanna Melesse - Department of Epidemiology and Biostatistics. Addis Continental Institute of Public Health, Addis Ababa, Ethiopia

Habtamu Wolde – Independent consultant. Addis Ababa, Ethiopia

Workagegnehu Tarekegn Kidane - Department of Nutrition and Behavioral Sciences. Addis Continental Institute of Public Health, Addis Ababa, Ethiopia

Yemane Berhane - Department of Epidemiology and Biostatistics. Addis Continental Institute of Public Health, Addis Ababa, Ethiopia

Sibylle Herzig Van Wees - Department of Global Public Health, Karolinska Institutet, Stockholm, Sweden

Corresponding author: Kalkidan Yibeltal

Postal address- 26751/1000, Addis Ababa, Ethiopia

Email- kalkidanyibeltal@addiscontinental.edu.et

Word count - 4119

For peer review only - http://bmjopen.bmj.com/site/about/guidelines.xhtml

Abstract

Objective- This study explored faith leaders' perspectives on the COVID-19 vaccine and their role in building COVID-19 vaccine trust in Addis Ababa, Ethiopia.

Design- A qualitative study with in-depth interviews and thematic analysis was conducted.

Participants- Twenty-one faith-leaders from the seven religious groups represented in the Inter-Religious Council of Ethiopia participated in the study.

Setting- The study was conducted in Addis Ababa, Ethiopia.

Results- The thematic analysis revealed three themes. Firstly, faith-leaders were aware of the risks of the COVID-19 pandemic, although most ascribed a spiritual meaning to the advent of the pandemic. The pandemic seriously affected the faith communities, inflicting financial losses. Secondly, faith-leaders were essential allies during the pandemic by effectively collaborating with government and health professionals in COVID-19 prevention activities and public health interventions using spiritual reasoning. They were actively informing the community about the importance of the COVID-19 vaccine, where many faith-leaders were publicly vaccinated to build trust in the vaccine and be role models. Thirdly, despite this, they faced multiple questions from the congregation about the vaccine, including rumors.

Conclusions- This research showed that faith-leaders played crucial roles in encouraging vaccine use but were limited in their persuasion power because of intense rumors and misinformation. Empowering faith-leaders with the latest vaccine evidence needs to be prioritized in the future.

Keywords: COVID-19, faith-leaders, vaccine, hesitancy, Ethiopia, religion

Strengths and limitations of this study

- Prolonged engagement with data was ensured as the co-authors conducted the interviews.
- Working with multiple coders aided in minimizing the subjective bias of any researchers.
- Interviews were conducted among faith-leaders included in the structured inter-religious council; it did not directly interview those outside of this structure to capture their perception and role

For peer review only - http://bmjopen.bmj.com/site/about/guidelines.xhtml

Background

Equitable access to safe and effective vaccines is critical to control the spread of infectious pandemics such as COVID-19 and minimize the devastating adverse health outcomes (1). Achieving herd immunity using effective vaccines is essential to avert pandemic-related morbidity and mortality (2,3). Promoting the uptake of COVID-19 vaccines requires people's willingness to get vaccinated and ensuring proper communication of information through trusted sources to aid vaccine acceptance (4,5).

Ethiopia started the COVID-19 vaccination campaign in March 2021 with the Astra Zeneca vaccine, manufactured by the Serum Institute of India and supplied by the COVID-19 Vaccines Global Access (COVAX) initiative (6). The Ministry of Health launched a national ceremony to initiate the COVID-19 vaccine rollout in the presence of different stakeholders, including religious representatives. Initially, healthcare providers and older people with comorbidities were prioritized and given two doses of AstraZeneca vaccines (6,7). In the later stage, the Johnson & Johnson vaccine, Sinopharm, and Pfizer vaccines were included in the campaign as they became available (7,8).

Vaccines need to be effectively utilized by the target population to bring about the intended effect of controlling the pandemic. However, despite the availability of vaccines and vaccination services, vaccine hesitancy- refusal or delay to accept a vaccine, has become a significant concern (9,10). WHO considers vaccine hesitancy as one of the top 10 Global Health threats (11). Concerns about vaccine safety, efficacy, and side effects have driven vaccine hesitancy related to COVID-19 worldwide (12,13). Moreover, the slow rollout and politicization of vaccines in Africa have fueled rumors, doubts, and conspiracy theories about these vaccines (14,15). Lack of trust in the government and the private sector, reading misinformation on social media, and the history of vaccination programs in a country have been further reasons for vaccine hesitancy among health workers and the population in Africa (13,14,16,17). Studies have shown high COVID-19 vaccine hesitancy among the Ethiopian population and some factors mentioned for the hesitancy were having social media as a primary information source, fear of side effects of the vaccine and thinking it as a biological weapon and doubts about the vaccine (15,18–20).

Global surveys show that religious factors are the third most commonly reported reason for vaccine hesitancy globally (21). Religious views can affect how people react to vaccinations, causing

For peer review only - http://bmjopen.bmj.com/site/about/guidelines.xhtml

reactions like vaccine hesitancy despite conveying medically sound and scientifically proven information/evidence (22). In the context of the COVID-19 pandemic, concerns have been raised that religious communities may serve as the basis for misinformation and unfounded theories that undermine the use of COVID-19 vaccines (23). Thus, the importance of faith-leaders in addressing vaccine hesitancy has received increasing attention in global health research (24).

Ethiopia is a religious country where more than 97% religious population, and faith-leaders hold significant societal positions (25). It has been well documented that faith-leaders are embedded in society with high-influencing positions and often have considerable leverage with state and non-state powers due to the size of their constituencies (26–29). Therefore, engagement with faith-leaders is critical as they are known to be gatekeepers to local communities, with considerable influence on their communities' beliefs and behavior. Thus, this study aimed to explore faith leaders' perspectives on the COVID-19 vaccine and the role faith-leaders can play in building COVID-19 vaccine trust.

Methods

Study setting

This study was conducted in Addis Ababa, the capital city of Ethiopia. Orthodox Christianity, Islam, and Protestantism are the main religions in Ethiopia (25). The Inter-Religious Council of Ethiopia (IRCE) was established in 2010 to promote inter-religious harmony in Ethiopia (30). IRCE is represented at federal, regional, and district levels. The IRCE head office is located in Addis Ababa. This council comprises seven religious groups, each with a representative in the head office.

Study design and aim

A qualitative study design using in-depth interviews was used to explore faith leaders' perceptions of the COVID-19 vaccine and the role faith-leaders can play in building COVID-19 vaccine trust.

Study population

The participants of this study are faith-leaders selected from the seven religious groups represented in the IRCE, which comprises Ethiopian Orthodox Tewahido Church, Islam, 7th Day Adventist, Evangelical churches fellowship of Ethiopia, Ethiopian Kale Heywet church, Ethiopian Evangelical Church Mekane Eyesus, and Ethiopian Catholic church.

For peer review only - http://bmjopen.bmj.com/site/about/guidelines.xhtml

Study sample and sampling procedure

Purposive sampling was used to identify appropriate study participants. The purpose of reaching out to the inter-religious council was with the notion that all the country's major religions were represented, and interviewing these participants would provide us with a comprehensive understanding of the country's context. We identified the seven religious entities constituting the IRCE through the inter-religious council. From these seven religions, informative individuals were selected in consultation with the head office representatives of each religion. We selected three study respondents from each religious entity: two faith-leaders at the head offices and one from the religious institutions (Churches and Mosques), culminating in 21 participants. The later interviews in the study did not generate new information, so we assumed reaching data saturation. The data collection was conducted over a period of two months.

Data collection procedures

In-depth interviews (IDI) were used for data collection. The themes of the interview guide were developed based on our background research. Themes included faith-leaders awareness of the COVID-19 and its risks, rumors and uncertainty around the COVID-19 vaccine, and the role of faith-leaders in controlling the pandemic. The research team developed the interview guide in English and translated it into Amharic (the official language). Two research assistants with extensive experience in qualitative data collection were recruited to collect the data. The research assistants were trained for two days on the interview guides, the purpose of the study, and research ethics. Verbal consent was obtained before the interview. All interviews were audio recorded, and additional notes were taken during the in-depth interview. The IDIs were conducted in a private and quiet place, mainly in an area preferred by the participants, their offices most of the time. The interviews took 40 - 90 minutes and were conducted on dates and times convenient to the participants. COVID-19 prevention methods such as keeping physical distance, using hand sanitizers, and wearing masks were strictly implemented during the data gathering.

Data analysis

The research assistants transcribed all interviews verbatim and then translated from Amharic to English. Transcripts and translations were checked for accuracy and consistency by the two independent persons in the research group who did not conduct the interviews. Field notes taken during the IDIs were integrated into the transcription. KY, FW, and SHvW conducted a thematic

For peer review only - http://bmjopen.bmj.com/site/about/guidelines.xhtml

qualitative analysis following Braun and Clarke's method (31). All data was coded double blinded. After one round of coding, the coding team met to develop a codebook and kept all audit trail records, such as data reduction notes. The codebooks included definitions and example responses. A further round of coding, using the codebook, was completed. The coding process was continued until the data was exhausted. Two further meetings were held to compare categories. Three more meetings, both in person and virtually, were made to create the themes initially by the coding team and then by the full team. Careful coding and multiple meetings ensured consistency and validity of the data analysis process. Working with multiple coders aided in minimizing the subjective bias of researchers. The Thematic analysis was done using Atlas.ti software version 7.5.16.

Patient and public involvement

No patient is involved.

Ethical considerations

Participation in this study was entirely voluntary, and informed consent was obtained from all participants. The study procedures, benefits, anticipated risks or discomforts of participating, and right to withdraw were explained. Principles of anonymity and confidentiality were applied to protect the participants' identities by assigning codes to them. The audio recordings were destroyed after the completion and verification of the transcription and translations. The study was conducted following national ethical standards and the declaration of Helsinki (32).

For peer review only - http://bmjopen.bmj.com/site/about/guidelines.xhtml

Results

The thematic analysis revealed three themes and 15 categories, Table 1. A category was created when many codes or data extracts described that specific finding. We only use one data extract to describe the category when presenting the data in this section.

Table 1 Themes and defined categories based on thematic analysis.

Theme	Category
Faith-leaders were aware of the risks of the COVID-19 pandemic, and they and their congregations were seriously affected by the pandemic.	Faith-leaders were aware of the risks of the COVID-19 pandemic.
	Solitude in difficult times
	Financial loss for faith institutions
	Vulnerable groups are left behind.
	Spiritual crisis
Faith-leaders were important allies during a pandemic	Faith-leaders were trusted
	Faith-leaders collaborated with government and health professionals.
	Faith-leaders engaged in public health prevention using spiritual reasoning.
	Faith-leaders trusted medical science.
	Faith-leaders were actively engaged in COVID-19 prevention methods.
Faith-leaders were intrigued by rumors and uncertainty around COVID-19 vaccine	Faith-leaders trusted vaccines
	Rumors about vaccines were rampant in the community.
	Spiritual rumors: 666
	Faith-leaders tried to address concerns.
	Faith-leaders were unsure how to respond to Rumors.

Faith-leaders were aware of the COVID-19, and they and their congregations had been seriously affected by the pandemic.

Although most faith-leaders in this study ascribed a spiritual meaning to the advent of the COVID-19 pandemic, all faith-leaders interviewed were aware of the risk and severity of the COVID-19 pandemic. They were well-informed and described in great detail the severe effects it had on their religious practice and the members of their spiritual community. In the words of a faith leader:

For peer review only - http://bmjopen.bmj.com/site/about/guidelines.xhtml

"There is nothing it did not touch; it has affected our social life, economy, religious relations, our office, our work... We have lost so many people." (IDI-12)

Faith-leaders described the implications of solitude under challenging times, particularly during times of mourning and grief. Participants expressed profound sadness that they were unable to console individuals and support them during times of grief: *"We could not even comfort the families of the deceased after the funeral. We consider spiritual support as a psychosocial treatment, but we failed to offer that too."* (IDI-4)

Moreover, faith-leaders described a spiritual crisis in their institutions whereby relationships between faith-leaders and those attending religious institutions had been harmed. This is mainly due to not allowing people to participate in religious services, which led to conflicts in several religious institutions. Faith-leaders described a change in spiritual practice and organization, which has remained in practice:

> *It* [COVID-19] *even created another culture; older people and people with some medical conditions became too scared to come to our congregation even after we were allowed to gather again. In our religion, Christianity, the New Testament encourages and teaches togetherness to grow in holiness. So, it affected that core value.* (IDI-6)

Faith-leaders further described the financial implications for their institutions. Due to the loss of participants in religious services, income diminished substantially. Businesses surrounding religious institutions were further negatively affected. This led to significant staff cuts at religious institutions. Moreover, it led to the closure of social activities and programs used to serve the needs of the poorest: *"With the limited money we got, we were only able to pay salaries church ministers; there was nothing left to give to those who are in need."* (IDI-4)

Despite these challenges, faith-leaders described innovative approaches to countering challenges. This includes the use of social media to reach the community:

> *We tried to solve this and reach our members through technology by calling them over the phone and using the Zoom application. Fortunately, we launched a Television program a month or two before the pandemic called Hope Channel, and we used that to preach and praise the Lord...* (IDI-14)

For peer review only - http://bmjopen.bmj.com/site/about/guidelines.xhtml

Active fundraising in the community, including collecting donations from individuals and businesses in the area, was further organized to make up for the financial loss:

> *When everyone was told to stay home, it was hard for our community because they had no money or food to eat, especially during fasting. So, we have tried our best to provide them food for the fasting period. We did that here in Addis Ababa and in some other areas, too. For that, we have collected over 30 million birr from investors and distributed it to others.* (IDI-16)

Faith-leaders are important allies during a pandemic.

The second theme summarizes faith-leaders and their institutions' work during the pandemic. To begin with, most faith-leaders collaborated closely with the Ministry of Health and health professionals to inform their congregations about the disease and preventive measures. The collaboration between the government and religious institutions appears to have been effective, and faith-leaders trusted the information and directives of the Ministry of Health:

> *"In our church, I invited different medical doctors to convey health messages about getting vaccinated and safety measures practices recommended by the health ministry and the government. Based on these messages, I encourage people to practice what they have heard."* (IDI-20)

In turn, faith-leaders consider themselves highly trusted within their communities and congregations. They are consulted in times of hardship and on important family matters.

> *"Religious leaders have many roles not only in controlling pandemics but also in other national affairs. This is because many people trust religious leaders; they obey them. Their word is associated with God, and what they say will get higher acceptance. Instead of a soldier speaking with a weapon, a priest holding a cross is more heard and accepted. This is a well-known fact in our country"* (IDI-2).

They were also consulted about questions regarding the COVID-19 pandemic, mainly rumors about the origin of the pandemic and vaccines. There are numerous examples of how faith-leaders describe following directives, implementing recommended interventions (hand washing, mask use, social distancing), and actively informing the community about importance of the COVID-19

For peer review only - http://bmjopen.bmj.com/site/about/guidelines.xhtml

vaccine.

> *Questions are forwarded to me; we often convey a message when we finish teaching in the church. That people should follow the instructions. In general, we tell them God loves us as we obey, wash our hands, we should respect each other, and the vaccine is also important you should take it. What we do here is critical; we are spiritual doctors.* (IDI-3)

Faith-leaders saw themselves as key in implementing measures to address the pandemic and proactively raising awareness to help implement directives.

> *We intensively created awareness, stressing that the vaccine is one of the major remedies for this disease. We assigned responsible persons and Imams who can transmit these messages during the five prayer times in every Mosque. Regarding awareness creation, we successfully changed people's understanding from wrong to right direction.* (IDI-16)

Faith-leaders described how they used religious scriptures to promote guidelines to reduce transmission. This was particularly important when the community challenged them regarding these measures.

> *The Bible supports all the recommended preventive safety measures for COVID-19. So, if parishioners come with such concerns, I will teach them this according to the Bible and guide them in the right direction.* (IDI-2)

Several faith leaders, regardless of the denomination, described examples of how they used spiritual reasoning to address concerns: *...the Bible says that Luke, the Evangelist, was one of the writers of the Bible who had medical knowledge. Therefore, the doctrine of the Church does not contradict such wisdom.* (IDI-11) Almost all faith-leaders in this study described a strong trust in science and use religious reasoning to support this. In the words of a faith leader*: "God protects us from death through faith and science"* (IDI-21)

Faith-leaders are faced with rumors and uncertainty around the COVID-19 vaccine.

A significant challenge described by faith-leaders was the rumors about the COVID-19 vaccine they encountered. This section summarizes faith leaders' different engagements with these rumors. Despite the strong commitment of faith-leaders to implement preventative measures, they faced challenges in addressing rumors about vaccines. Most, but not all, faith-leaders in this study trust

For peer review only - http://bmjopen.bmj.com/site/about/guidelines.xhtml

the COVID-19 vaccine. They described their trust in science and the government and used religious reasoning to support their trust. Many faith-leaders were publicly vaccinated to build trust in the vaccine: *"I and other faith-leaders of different religions in Addis Ababa got vaccinated in public; we did that to be role models to our parishioners."* (IDI-13)

Participants described numerous and consistent rumors encountered in the community. The COVID-19 vaccine was considered dangerous because it was sent from abroad and was therefore not trusted: *"Now Ethiopia is in disagreement with America as we all know, what if they send this vaccine to destroy us and attack us?"* (IDI-14). There were rumors that the COVID-19 vaccine represented 666 – the mark of the beast.

> *There is a religious teaching about 666*[1]*. I guess you have heard about that in every church. So, they say this vaccine is used to insert satanic 666 into our bodies. So, they say Satan makes the vaccine, so this is satanic teaching. They say participating in this is like partnering with Satan.* (IDI-13)

Moreover, a repeated concern that faith-leaders faced, and some addressed themselves, was the effect of the COVID-19 vaccine on fertility. Faith-leaders described how rumors affected their own beliefs, and they described challenges in addressing these questions:

> *..it is rumored that getting vaccinated can cause infertility. They also say that developed countries fabricate this disease to minimize the population of highly populated countries like African countries. There were so many questions. It was tough to answer such questions.* (IDI-16)

Few faith-leaders actively countered the rumors, but those who did use spiritual reasoning to do so. For example:

> *When they say it will make you infertile, I will say, are you a prophet? Have you been given the gift of prophesying? So this shows an awareness problem due to a lack of awareness; that's how I see it.* (IDI-3)

However, several faith-leaders described that they got uncertain about the vaccine as a result of the rumors: "*It's best to try to understand what they want to say. I don't have evidence it might be what they said or it may not. So the main thing is believing."* (IDI-21)

---

[1] *According to most manuscripts of the New Testament and in English translations of the Bible, the number of the beast is six hundred sixty-six. (Book of Revelation 13:15-18, Wikipedia)*

For peer review only - http://bmjopen.bmj.com/site/about/guidelines.xhtml

Moreover, some described that they could not address those concerns. They were more comfortable preaching about all other public health prevention measures than the vaccine.

> *One of our [faith-leaders] tasks is to educate the community on preventing these outbreaks as health professionals recommended. Teaching about the disease/ pandemic is comfortable, but I cannot say anything about specific vaccines.* (IDI-11)

For peer review only

For peer review only - http://bmjopen.bmj.com/site/about/guidelines.xhtml

Discussion

This qualitative study explored faith leaders' role in combating the pandemic and in building COVID-19 vaccine trust in Addis Ababa. The findings of our study showed that most faith-leaders included in this study were aware of the risks associated with COVID-19. Due to the advent of the COVID-19 pandemic, faith-leaders and their congregations have faced solitude in challenging times. Additionally, religious institutions have encountered significant financial and spiritual crises. Our study also revealed that faith-leaders were actively engaged in pandemic measures and held strong beliefs in the medical sciences, including vaccines. However, faith-leaders struggled to address COVID-19 vaccine rumors in their communities despite the high trust in vaccines and medical science.

This study indicated that faith-leaders were well-informed about the risks of COVID-19. This could be due to reasonable access to sources regarding the COVID-19 pandemic due to their networks, mass media, and social media exposure. Our findings disclosed that due to the COVID-19 pandemic, vulnerable groups, faith leaders, and their congregations have faced solitude during challenging times and significant financial and spiritual crises. This finding aligns with a qualitative study conducted in Ghana, which showed that the COVID-19 pandemic caused economic challenges to the congregants, a decline in spiritual life, and a loss of fellowship and community (33). In another qualitative study conducted on United Methodist pastors in one of the two Annual Conferences in North Carolina, Pastors reported that COVID-19 fundamentally unsettled the routine works of the ministry (34).

This study also identified innovative approaches faith-leaders use to tackle the challenges. This includes using social media to reach the community and fundraising to compensate for the financial loss. A qualitative study on Ghanaian Christian Leaders also showed that as a response to the ban, most participants in their research explicitly reported that they moved at least some activities online, which included conventional activities, such as posting prayer topics and live streaming of services for audiences who wished to view them synchronously (35). Similarly, religious authorities in Uganda played a critical role in delivering public health messages on COVID-19 risk communication using the web .

According to a recent systematic review that assessed religious communities' role, religion has acted as an essential platform for inter-sectoral collaboration with science and government to

For peer review only - http://bmjopen.bmj.com/site/about/guidelines.xhtml

combat COVID-19 (36). National, regional, and local faith-leaders have high levels of influence and community-organizing capabilities. Faith-leaders are effective messengers endorsing vaccination compared to other potential messengers (28). They can help frame approaches that make them more likely to be accepted in their communities. Governments should build trust with faith-leaders and integrate them into planning, decision-making, and implementation at every level of their COVID-19 response (37). The findings in this study also showed that there was a strong collaboration between the government and religious institutions, which appeared to have been effective, and faith-leaders trusted the information and directives of the Ministry of Health. This might be due to the closeness of the religious institutions to each other and the government through a well-founded and organized inter-religious council that collaborates with the government on different national agendas (30). Besides, all faith-leaders interviewed for this study consider themselves highly trusted within their communities and congregations. They were consulted about questions regarding the COVID-19 pandemic, mainly rumors about the origin of the pandemic and vaccines. Due to that, most of the study participants saw themselves as critical actors in implementing the safety measures and in proactively raising awareness to help implement directives to address the pandemic. Since faith-leaders are often highly influential community leaders who are 'listened to by the community members, they often underutilize the potential for catalyzing change (24). In line with this finding, a qualitative case study conducted in Indonesia showed that the faith-leaders supported the health directives designed to reduce high transmission risk (38). All the scholars and faith-leaders stated that funerals, according to the health protocols issued by the authorities, should be conducted considering potential disease transmission. Some Islamic organizations have developed their guidelines for COVID-19 by modifying the religious values of certain institutions to prevent the spread of infectious diseases (38).

This study revealed that regardless of the denomination, almost all faith-leaders who participated in this study said they trust in science and use religious scriptures and spiritual reasoning to promote guidelines to reduce the transmission of COVID-19. A study has also shown that many faith-leaders see the vaccine as a message of hope. Pope Francis also indicated everyone's moral obligation to be vaccinated (39). Our study participants added that many faith-leaders were publicly vaccinated to build trust in the vaccine. This was particularly important when the community challenged them regarding these measures. In agreement with that, findings of a qualitative study conducted in Leeds, United Kingdom, also revealed that faith-leaders could play

For peer review only - http://bmjopen.bmj.com/site/about/guidelines.xhtml

a vital role in the health behavior of their congregants (27). Faith-leaders can influence health behavior not only on the individual but also on a sociocultural and environmental level. They exert such influence through several mediators, including as scriptural power, social influencer, and serving as role models (27). Our results showed most of the faith-leaders faced numerous and consistent rumors in the community. They were affected by the rumors they heard about the COVID-19 vaccine and faced challenges in addressing such questions.

The African Center for Strategic Studies reported that the preponderance of COVID-19 vaccine myths is causing many Africans to forego vaccinations when new, more transmissible coronavirus variants spread across the continent (40). The report indicated several myths, including live viruses are injected to cause death after vaccination; the vaccine causes infertility by altering the DNA to reduce Africa's population, and other serious side effects that are worse than contracting the virus, and some claim that vaccines are a cover to implant traceable microchips (40). Such myths and similar rumors may lead to COVID-19 vaccine hesitancy in the general community. In line with these findings, a population-based online survey conducted in Ethiopia showed that only 31.4% of participants were willing to receive the COVID-19 vaccine (19). One of the strategies to counter vaccine hesitancy is to follow a multisectoral approach that involves the collaboration between various stakeholders, such as government, private companies, religious groups, and other agencies, to leverage the knowledge, expertise, and resources, thereby enabling the creation of longstanding public trust of vaccines (27,39). Given the importance of faith-leaders and their trust in science, equipping faith-leaders with the knowledge and skills to address rumors appears indispensable.

Limitations of the study can lay in the fact that we only interviewed faith-leaders included in the structured inter-religious council and did not directly interview those outside of this structure to capture their perception and role. Although the findings cannot be generalized, inferential generalizations can be drawn, and results may be relevant for other similar settings.

Conclusion

Faith-leaders have played an essential role in mitigating the COVID-19 pandemic in Ethiopia. Faith-leaders have followed and implemented government directives and promoted the vaccine, further amplifying their important alliance in public health. However, they have been challenged with different rumors about the vaccine, which they struggled to address as they were not equipped to do so. Thus, empowering faith-leaders to address rumors about new vaccines could have

For peer review only - http://bmjopen.bmj.com/site/about/guidelines.xhtml

significant public health implications for ongoing and future pandemics. Future studies need to consider involving faith leaders at all levels and to utilize quantitative methods. In addition, intervention studies addressing issues of hesitancy are necessary.

Contributor statement – YB, SHvW, FW, and KY designed the study. HM & HW conducted the interviews, transcriptions, and translations. KY, FW, HM, WT, and SHvW were involved in the coding and conducted the analysis. All authors collaborated on the writing of the manuscript, and the final draft was written by KY & FW. All authors have read and approved the final manuscript.

Competing Interests - No conflicts of interest to declare.

Funding – The Addis Continental Institute of Public Health, Ethiopia, funded the study. SHvW is the recipient of a Postdoc grant awardee by FORTE (STY396 2021/0001) in Sweden.

Data sharing statement – Data are available upon reasonable request

Ethics approval- Ethical clearance was obtained from the Institutional Review Board (IRB) of Addis Continental Institute of Public Health (ACIPH/IRB/010/2021).

Acknowledgments – We would like to acknowledge the inter-religious council of Ethiopia for their collaboration for the study.

For peer review only

Reference

1. Legge D, Kim S. Equitable Access to COVID-19 Vaccines: Cooperation around Research and Production Capacity Is Critical. 2021 Jul 27; Available from: https://opal.latrobe.edu.au/articles/journal_contribution/Equitable_Access_to_COVID-19_Vaccines_Cooperation_around_Research_and_Production_Capacity_Is_Critical/15050310/1

2. Bardosh K, Figueiredo A de, Gur-Arie R, Jamrozik E, Doidge J, Lemmens T, et al. The unintended consequences of COVID-19 vaccine policy: why mandates, passports and restrictions may cause more harm than good. BMJ Glob Health. 2022 May 1;7(5):e008684.

3. Pilkington V, Keestra SM, Hill A. Global COVID-19 Vaccine Inequity: Failures in the First Year of Distribution and Potential Solutions for the Future. Front Public Health. 2022 Mar 7;10:821117.

4. World Bank. Accelerating COVID-19 Vaccine Deployment : Removing Obstacles to Boost Coverage Levels and Protect Those at High-risk (English). Available from: https://documents.worldbank.org/en/publication/documents-reports/documentdetail

5. Druedahl LC, Minssen T, Price WN. Collaboration in times of crisis: A study on COVID-19 vaccine R&D partnerships. Vaccine. 2021 Oct 8;39(42):6291–5.

6. WHO | Regional Office for Africa. 2023. Ethiopia introduces COVID-19 vaccine in a national launching ceremony. Available from: https://www.afro.who.int/news/ethiopia-introduces-covid-19-vaccine-national-launching-ceremony

7. COVAX | MINISTRY OF HEALTH - Ethiopia. Available from: https://www.moh.gov.et/site/covax

8. The Ministry of Health has received 10 million doses of covid 19 vaccine from the Chinese government. | MINISTRY OF HEALTH - Ethiopia . Available from: https://www.moh.gov.et/site/node/352

9. Mesch GS, Schwirian KP. Vaccination hesitancy: fear, trust, and exposure expectancy of an Ebola outbreak. Heliyon. 2019 Jul 18;5(7):e02016.

10. Sallam M. COVID-19 Vaccine Hesitancy Worldwide: A Concise Systematic Review of Vaccine Acceptance Rates. Vaccines. 2021 Feb 16;9(2):160.

11. Scheres J, Kuszewski K. The Ten Threats to Global Health in 2018 and 2019. A welcome and informative communication of WHO to everybody. Zdr Publiczne Zarządzanie. 2019 Jan 1;17:2–8.

12. Solís Arce JS, Warren SS, Meriggi NF, Scacco A, McMurry N, Voors M, et al. COVID-19 vaccine acceptance and hesitancy in low- and middle-income countries. Nat Med. 2021;27(8):1385–94.

13. Biswas MdR, Alzubaidi MS, Shah U, Abd-Alrazaq AA, Shah Z. A Scoping Review to Find Out Worldwide COVID-19 Vaccine Hesitancy and Its Underlying Determinants. Vaccines. 2021 Oct 25;9(11):1243.

14. Ekwebelem OC, Onyeaka H, Yunusa I, Miri T, Onwuneme YM, Eunice AT, et al. Do we trust the government? Attributes of COVID-19 vaccine hesitancy and acceptance in Nigeria. AIMS Med Sci. 2022;9(2):268–82.

For peer review only

For peer review only - http://bmjopen.bmj.com/site/about/guidelines.xhtml

15. Dereje N, Tesfaye A, Tamene B, Alemeshet D, Abe H, Tesfa N, et al. COVID-19 vaccine hesitancy in Addis Ababa, Ethiopia: a mixed-method study. BMJ Open. 2022 May;12(5):e052432.

16. Ekwebelem OC, Nnorom-Dike OV, Aborode AT, Ekwebelem NC, Aleke JC, Ofielu ES. Eradication of wild poliovirus in Nigeria: Lessons learnt. Public Health Pract. 2021 May 15;2:100144.

17. Jegede AS. What Led to the Nigerian Boycott of the Polio Vaccination Campaign? PLOS Med. 2007 Mar 20;4(3):e73.

18. Terefa DR, Shama AT, Feyisa BR, Desisa AE, Geta ET, Cheme MC, et al. COVID-19 Vaccine Uptake and Associated Factors Among Health Professionals in Ethiopia. Infect Drug Resist. 2021;14:5531.

19. Belsti Y, Gela YY, Akalu Y, Dagnew B, Getnet M, Abdu Seid M, et al. Willingness of Ethiopian Population to Receive COVID-19 Vaccine. J Multidiscip Healthc. 2021;14:1233–43.

20. Mohammed R, Nguse TM, Habte BM, Fentie AM, Gebretekle GB. COVID-19 vaccine hesitancy among Ethiopian healthcare workers. PLOS ONE. 2021 Dec 17;16(12):e0261125.

21. USAID MOMENTUM. Effects of Faith Actor Engagement on the Uptake and Coverage of Immunization in Low- and Middle-Income Countries. Available from: https://usaidmomentum.org/resource/vaccine-hesitancy-report/

22. Garcia LL, Yap JFC. The role of religiosity in COVID-19 vaccine hesitancy. J Public Health Oxf Engl. 2021 Jun 3;fdab192.

23. Lee Rogers R, Powe N. COVID-19 Information Sources and Misinformation by Faith Community. Inq J Med Care Organ Provis Financ. 2022 May 30;59:00469580221081388.

24. Bartelink B, le Roux E, Palm S. Sleeping Giants: Mobilizing faith leaders as agents of change. London: Tearfund; 2017.

25. The DHS Program - Ethiopia: DHS, 2016 - Final Report (English). Available from: https://dhsprogram.com/publications/publication-fr328-dhs-final-reports.cfm

26. Anshel M, Smith M. The Role of Religious Leaders in Promoting Healthy Habits in Religious Institutions. J Relig Health. 2013 Mar 21;53.

27. Heward-Mills NL, Atuhaire C, Spoors C, Pemunta NV, Priebe G, Cumber SN. The role of faith leaders in influencing health behaviour: a qualitative exploration on the views of Black African Christians in Leeds, United Kingdom. Pan Afr Med J. 2018;30:199.

28. Viskupic F, Wiltse D. The Messenger Matters: Religious Leaders and Overcoming COVID-19 Vaccine Hesitancy. 2021.

29. Syed U, Kapera O, Chandrasekhar A, Baylor BT, Hassan A, Magalhães M, et al. The Role of Faith-Based Organizations in Improving Vaccination Confidence & Addressing Vaccination Disparities to Help Improve Vaccine Uptake: A Systematic Review. Vaccines. 2023 Feb;11(2):449.

30. Inter-Religious Council of Ethiopia. 2013. Available from: https://www.uri.org/who-we-are/cooperation-circle/inter-religious-council-ethiopia

For peer review only

For peer review only - http://bmjopen.bmj.com/site/about/guidelines.xhtml

31. Braun V, Clarke V. Using thematic analysis in psychology. Qual Res Psychol. 2006 Jan 1;3:77–101.

32. World Medical Association. World Medical Association Declaration of Helsinki: Ethical Principles for Medical Research Involving Human Subjects. JAMA. 2013 Nov 27;310(20):2191–4.

33. Osei-Kuffour F, Kwasi Peprah W, Marfo Sarfo D, Osei Yeboah B. COVID-19 Impact on Church Cash Inflows in Ghana as Moderated by Location Profile. Appl Finance Account. 2022 Oct 13;8(1):6.

34. Johnston EF, Eagle DE, Headley J, Holleman A. Pastoral Ministry in Unsettled Times: A Qualitative Study of the Experiences of Clergy During the COVID-19 Pandemic. Rev Relig Res. 2022;64(2):375–97.

35. Adams G, Osei-Tutu A, Affram AA, Phillips-Kumaga L, Dzokoto VAA. Implications of COVID-19 Innovations for Social Interaction: Provisional Insights From a Qualitative Study of Ghanaian Christian Leaders. Front Psychol. 2022 May 24;13:647979.

36. Lee M, Lim H, Xavier MS, Lee EY. “A Divine Infection”: A Systematic Review on the Roles of Religious Communities During the Early Stage of COVID-19. J Relig Health. 2022;61(1):866–919.

37. Tony-Blair-Institute-Working-With-Religious-Leaders-to-Support-Public-Health-Measures.pdf. Available from: https://docs.google.com/viewerng/viewer?url=https://jliflc.com/wp-content/uploads/2020/05/Tony-Blair-Institute-Working-With-Religious-Leaders-to-Support-Public-Health-Measures.pdf

38. Nurhayati N, Purnama TB. Funeral Processes During the COVID-19 Pandemic: Perceptions Among Islamic Religious Leaders in Indonesia. J Relig Health. 2021;60(5):3418–33.

39. Galang JRF. Science and religion for COVID-19 vaccine promotion. J Public Health Oxf Engl. 2021 Sep 22;43(3):e513–4.

40. Studies the AC for S. Dispelling COVID Vaccine Myths in Africa. Africa Center for Strategic Studies. Available from: https://africacenter.org/spotlight/dispelling-covid-vaccine-myths-in-africa/

For peer review only - http://bmjopen.bmj.com/site/about/guidelines.xhtml

Consolidated criteria for reporting qualitative studies (COREQ): 32-item checklist

Please indicate in which section each item has been reported in your manuscript. If you do not feel an item applies to your manuscript, please enter N/A.

For further information about the COREQ guidelines, please see Tong *et al.*, 2017: https://doi.org/10.1093/intqhc/mzm042

No.	Item	Description	Page #
**Domain 1: Research team and reflexivity**			
Personal characteristics			
1.	Interviewer/facilitator	Which author/s conducted the interview or focus group?	15
2.	Credentials	What were the researcher's credentials? *E.g. PhD, MD*	PhD, MPH, MD
3.	Occupation	What was their occupation at the time of the study?	Professor, Post doctoral researcher, Assistant Professors, Lecturer and research associate
4.	Gender	Was the researcher male or female?	Kalkidan, Firehiwot, Workagnehu, Hanna are females whereas Yemane and Habtamu are males
5.	Experience and training	What experience or training did the researcher have?	Minimum of 5 years and above experience on research and training
Relationship with participants			
6.	Relationship established	Was a relationship established prior to study commencement?	N/A
7.	Participant knowledgeof the interviewer	What did the participants know about the researcher? *E.g. Personal goals, reasons for doing the research*	N/A
8.	Interviewer characteristics	What characteristics were reported about the interviewer/facilitator? *E.g. Bias, assumptions, reasons and interests in the research topic*	N/A
**Domain 2: Study design**			
Theoretical framework			
9.	Methodological orientation and theory	What methodological orientation was stated to underpin the study? *E.g. grounded theory, discourse analysis, ethnography, phenomenology, content analysis*	5
Participant selection			
10.	Sampling	How were participants selected? *E.g. purposive, convenience, consecutive, snowball*	5
11.	Method of approach	How were participants approached? *E.g. face-to-face, telephone, mail, email*	5
12.	Sample size	How many participants were in the study?	5
13.	Non-participation	How many people refused to participate or dropped out? What were the reasons for this?	N/A

For peer review only

Setting			
14.	Setting of data collection	Where was the data collected? *E.g. home, clinic, workplace*	5
15.	Presence of non-participants	Was anyone else present besides the participants and researchers?	5

For peer review only

16.	Description of sample	What are the important characteristics of the sample? *E.g. demographic data, date*	5
Data collection			
17.	Interview guide	Were questions, prompts, guides provided by the authors? Was it pilot tested?	5
18.	Repeat interviews	Were repeat interviews carried out? If yes, how many?	N/A
19.	Audio/visual recording	Did the research use audio or visual recording to collect the data?	5
20.	Field notes	Were field notes made during and/or after the interview or focus group?	5
21.	Duration	What was the duration of the interviews or focus group?	5
22.	Data saturation	Was data saturation discussed?	No
23.	Transcripts returned	Were transcripts returned to participants for comment and/or correction?	N/A
**Domain 3: analysis and findings**			
Data analysis			
24.	Number of data coders	How many data coders coded the data?	15
25.	Description of the coding tree	Did authors provide a description of the coding tree?	5 & 7
26.	Derivation of themes	Were themes identified in advance or derived from the data?	5
27.	Software	What software, if applicable, was used to manage the data?	5
28.	Participant checking	Did participants provide feedback on the findings?	N/A
Reporting			
29.	Quotations presented	Were participant quotations presented toillustrate the themes / findings? Was each quotation identified? *E.g. Participant number*	8,9,10 & 11
30.	Data and findings consistent	Was there consistency between the data presented and the findings?	12, 13 & 14
31.	Clarity of major themes	Were major themes clearly presented in the findings?	7
32.	Clarity of minor themes	Is there a description of diverse cases or discussion of minor themes?	N/A

When submitting your manuscript via the online submission form, please upload the completed checklist as a Figure/supplementary file.

If you would like this checklist to be included alongside your article, we ask that you upload the completed checklist to an online repository and include the guideline type, name of the repository, DOI and license in the *Data availability* section of your manuscript.

Developed from: Allison Tong, Peter Sainsbury, Jonathan Craig, Consolidated criteria for reporting qualitative research (COREQ): a 32-item checklist for interviews and focus groups, International Journal for Quality in Health Care, Volume 19, Issue 6, December 2007, Pages 349–357, https://doi.org/10.1093/intqhc/mzm042
